# Very low-depth whole-genome sequencing in complex trait association studies

**DOI:** 10.1093/bioinformatics/bty1032

**Published:** 2018-12-21

**Authors:** Arthur Gilly, Lorraine Southam, Daniel Suveges, Karoline Kuchenbaecker, Rachel Moore, Giorgio E M Melloni, Konstantinos Hatzikotoulas, Aliki-Eleni Farmaki, Graham Ritchie, Jeremy Schwartzentruber, Petr Danecek, Britt Kilian, Martin O Pollard, Xiangyu Ge, Emmanouil Tsafantakis, George Dedoussis, Eleftheria Zeggini

**Affiliations:** bty1032-aff1 Department of Human Genetics, Wellcome Sanger Institute, Wellcome Genome Campus, Hinxton, UK; bty1032-aff2 Institute of Translational Genomics, Helmholtz Zentrum München – German Research Center for Environmental Health, Neuherberg, Germany; bty1032-aff3 Wellcome Centre for Human Genetics, University of Oxford, Oxford, UK; bty1032-aff4 Department of Biomedical Informatics, Harvard Medical School, Boston, MA, USA; bty1032-aff5 Department of Nutrition and Dietetics, School of Health Science and Education, Harokopio University of Athens, Athens, Greece; bty1032-aff6 European Bioinformatics Institute, Wellcome Genome Campus, Hinxton, UK; bty1032-aff7 Anogia Medical Centre, Anogia, Greece

## Abstract

**Motivation:**

Very low-depth sequencing has been proposed as a cost-effective approach to capture low-frequency and rare variation in complex trait association studies. However, a full characterization of the genotype quality and association power for very low-depth sequencing designs is still lacking.

**Results:**

We perform cohort-wide whole-genome sequencing (WGS) at low depth in 1239 individuals (990 at 1× depth and 249 at 4× depth) from an isolated population, and establish a robust pipeline for calling and imputing very low-depth WGS genotypes from standard bioinformatics tools. Using genotyping chip, whole-exome sequencing (75× depth) and high-depth (22×) WGS data in the same samples, we examine in detail the sensitivity of this approach, and show that imputed 1× WGS recapitulates 95.2% of variants found by imputed GWAS with an average minor allele concordance of 97% for common and low-frequency variants. In our study, 1× further allowed the discovery of 140 844 true low-frequency variants with 73% genotype concordance when compared to high-depth WGS data. Finally, using association results for 57 quantitative traits, we show that very low-depth WGS is an efficient alternative to imputed GWAS chip designs, allowing the discovery of up to twice as many true association signals than the classical imputed GWAS design.

**Availability and implementation:**

The HELIC genotype and WGS datasets have been deposited to the European Genome-phenome Archive (https://www.ebi.ac.uk/ega/home): EGAD00010000518; EGAD00010000522; EGAD00010000610; EGAD00001001636, EGAD00001001637. The peakplotter software is available at https://github.com/wtsi-team144/peakplotter, the transformPhenotype app can be downloaded at https://github.com/wtsi-team144/transformPhenotype.

**Supplementary information:**

Supplementary data are available at *Bioinformatics* online.

## 1 Introduction

The contribution of low-frequency and rare variants to the allelic architecture of complex traits remains largely uncharted. Power to detect association is central to genetic studies examining sequence variants across the full allele frequency spectrum. Whole-genome sequencing (WGS)-based association studies hold the promise of probing a larger proportion of sequence variation compared to imputed genome-wide genotyping arrays. However, although large-scale high-depth WGS efforts are now underway (Brody *et al.*, 2017), comparatively high costs do not yet allow for the generalized transposition of the GWAS paradigm to high-depth sequencing. As sample size and haplotype diversity are more important than sequencing depth in determining power for association studies (Alex Buerkle and Gompert, 2013; Le and Durbin, 2011), low-depth WGS has emerged as an alternative, cost-efficient approach to capture low-frequency variation in large studies. Improvements in calling algorithms have enabled robust genotyping using WGS at low depth (4–8×), leading to the creation of large haplotype reference panels (1000 Genomes Project Consortium *et al.*, 2015; McCarthy *et al.*, 2016), and to several low-depth WGS-based association studies (Astle *et al.*, 2016; Tachmazidou *et al.*, 2017; UK10K Consortium *et al.*, 2015). Very low-depth (<2×) sequencing has been proposed as an efficient way to further improve the cost efficiency of sequencing-based association studies. Simulations have shown that in whole-exome sequencing (WES) designs, extremely low sequencing depths (0.1–0.5×) are effective in capturing single-nucleotide variants (SNVs) in the common (MAF > 5%) and low-frequency (MAF 1–5%) categories compared to imputed GWAS arrays (Pasaniuc *et al.*, 2012). The CONVERGE consortium demonstrated the feasibility of such approaches through the first successful case-control study of major depressive disorder in 4509 cases and 5337 controls (Converge Consortium, 2015), and we previously showed that 1× WGS allowed the discovery of burdens of low-frequency and rare variants that replicate in cosmopolitan and diverse populations (Gilly *et al.*, 2016). However, a systematic examination of genotyping quality from 1× WGS and its implications for power in association studies is lacking, posing the question of the generalizability of such results in the wider context of next-generation association studies. Here, we perform very low depth (1×), cohort-wide WGS in an isolated population from Greece, show that imputation tools commonly used with chip data perform well using 1× WGS, and establish a detailed quality profile of called variants. We then demonstrate the advantages of 1× WGS compared to the more traditional imputed GWAS design both in terms of genotype accuracy and power to detect association signals.

## 2 Results 

**Fig. 1.**
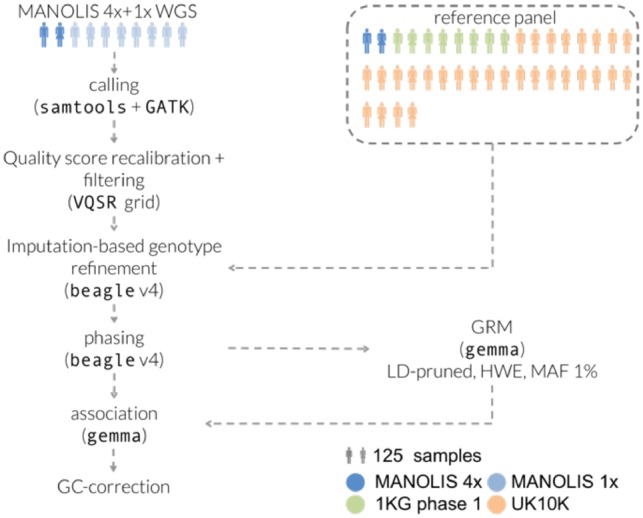
Processing pipeline for the MANOLIS 1× data. Tools and parameters for the genotype refinement and phasing steps were selected after benchmarking 13 pipelines involving four different tools (see Section 4)

As part of the Hellenic Isolated Cohorts (HELIC) study, we whole-genome sequenced 990 individuals from the Minoan Isolates (HELIC-MANOLIS) cohort at 1× depth, on the Illumina HiSeq2000 platform. In addition, 249 samples from the MANOLIS cohort were sequenced at 4× depth (Southam *et al.*, 2017). Imputation-based genotype refinement was performed on the cohort-wide dataset using a combined reference panel of 10 244 haplotypes from MANOLIS 4× WGS, the 1000 Genomes (1000 Genomes Project Consortium *et al.*, 2015) and UK10K (UK10K Consortium *et al.*, 2015) projects (Fig. 1).

### 2.1 Variant calling pipeline

Prior to any imputation-based refinement, our approach allowed the capture of 80 and 100% of low-frequency (MAF 1–5%) and common (MAF > 5%) SNVs, respectively, when compared to variants present on the Illumina OmniExpress and HumanExome chips genotyped in the same samples. In 10 control samples from the Platinum Genomes dataset (Eberle *et al.*, 2017) with high-depth WGS data (50×) downsampled to 1×, joint calling with MANOLIS resulted in pre-imputation false-positive and false-negative rates of 12 and 24.6%, respectively (see Section 4).

**Fig. 2.**
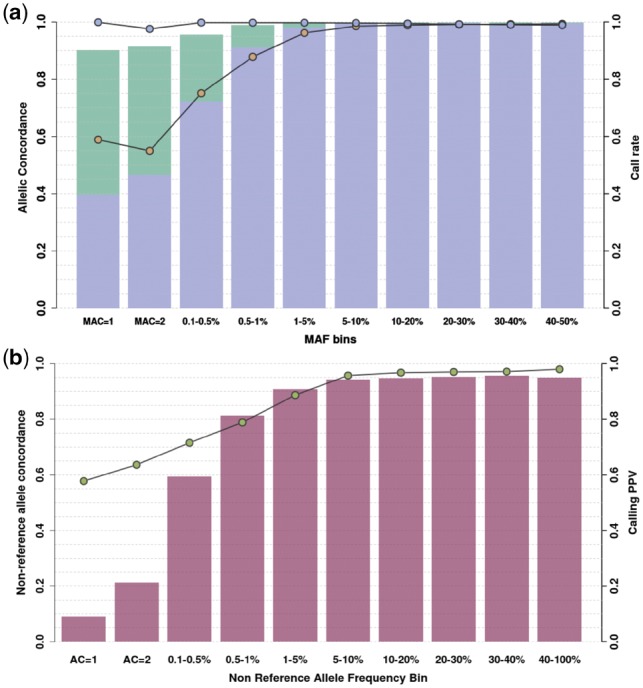
Concordance and call rate for low-depth WGS genotypes. (**a**) Genotype (blue circles) and minor allele (yellow circles) concordance is computed for 1239 samples in MANOLIS (4× and 1×) against merged OmniExpress and ExomeChip data. Call rate is assessed for the refined (purple) and refined plus imputed (green) datasets. (**b**) Non-reference allele concordance (green circles) and PPV (fuchsia bars) are computed for 1127 MANOLIS samples with both 22× WGS and low-depth calls

In order to improve sensitivity and genotype accuracy, we compared 13 genotype refinement and imputation pipelines using tools commonly used for genotyping chip imputation, using directly typed OmniExpress and ExomeChip genotypes as a benchmark (see Section 4). We used a reference panel containing haplotypes from 4873 cosmopolitan samples from the 1000 Genomes and UK10K projects, as well as the phased haplotypes from 249 MANOLIS samples sequenced at 4× depth. The best-performing pipeline, described in Figure 1, captures 95% of rare, 99.7% of low-frequency and 99.9% of common variants present in chip data, with an average minor allele concordance of 97% across the allele frequency spectrum (see Section 4, Fig. 2a, Supplementary Fig. S1 and Table S1). About 79.7% of 1× WGS variants were found using high-depth WGS at 22× in a subset of the MANOLIS samples (*n* = 1127), although this positive predictive value varied across the MAF spectrum, from 8.9% for singletons to 95.1% for common variants (Fig. 2b). Genotype concordance was similar, although slightly lower, when compared to the chip variants. Due to the 22× data being aligned to a different build and the unmappable regions resulting from a lift-over, we were unable to compute genome-wide false-positive rates, however by comparing 1× calls with those produced by WES in five individuals from the MANOLIS cohort, we estimate a false-positive rate of 2.4% post-imputation in the coding parts of the genome (see Section 4).

### 2.2 Comparison of variant call sets with an imputed GWAS

**Fig. 3.**
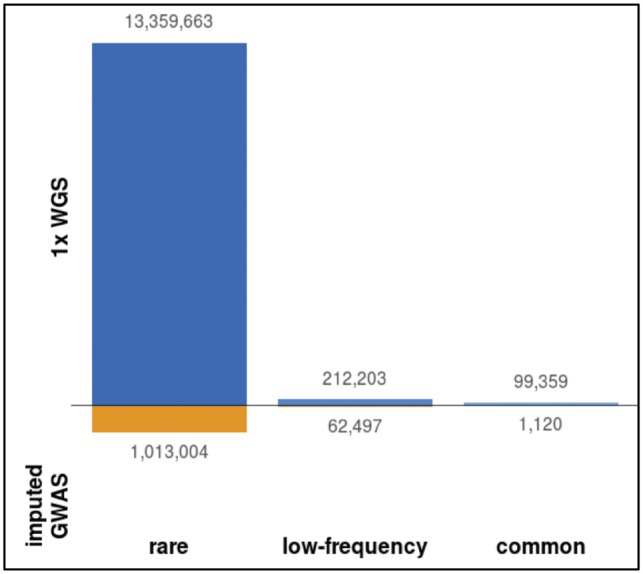
Unique variants called by sequencing and imputed GWAS. Variants unique to either dataset, arranged by MAF bin. Both datasets are unfiltered apart from monomorphics, which are excluded. MAF categories: rare (MAF<1%), low-frequency (MAF 1–5%), common (MAF>5%)

The genotype refinement and imputation step yielded 30 483 136 non-monomorphic SNVs in 1239 MANOLIS individuals. The number of variants discovered using 1× WGS is nearly twice as high as that from array-based approaches. In a subset of 982 MANOLIS individuals with both 1× WGS, OmniExpress and ExomeChip data, we called 25 673 116 non-monomorphic SNVs using 1× WGS data, compared to 13 078 518 non-monomorphic SNVs in the same samples with chip data imputed up to the same panel (Southam *et al.*, 2017) without any imputation INFO score filtering. The main differences are among rare variants (MAF < 1%) (Fig. 3): 13, 671, 225 (53.2%) variants called in the refined 1× WGS are absent from the imputed GWAS, 98% of which are rare. About 82% of these rare unique SNVs are singletons or doubletons, and therefore 9.5% of all variants called in the 1× WGS dataset were unique variants with minor allele count >2.

**Fig. 4.**
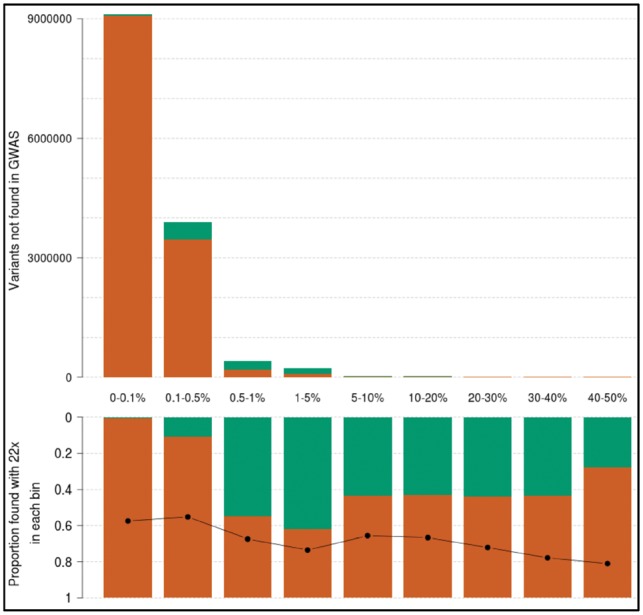
Frequency and positive predictive value of variants in 1× sequencing not found by GWAS and imputation; 1× variants not found in the GWAS data, arranged by MAF bin, in raw numbers (top). Green bars count variants recapitulated in the 22× (true positives). The proportion of these over the total (positive predictive value) is displayed in each bin in the bottom panel. The black line indicates minor allele concordance for true positive variants. The first category (0–0.1%) contains singletons and doubletons only

A crucial question is the proportion of true positives among these additional SNVs not found by GWAS and imputation. By comparing their positions and alleles with high-depth WGS in the same samples, we find that the positive predictive value (PPV) profile for these variants is much lower compared to when all variants are examined (Figs 4 and 2b). As expected, PPV is almost zero for additional singletons and doubletons, and just above 40% for the few additional common variants. About 62% of low-frequency variants unique to the 1× are true positives, which correspond to 140 844 low-frequency variants with high genotyping quality that are missed by the imputed GWAS. Minor allele concordance is lower than for all variants, with a lower bound at 55% for rare variants and reaching 73% for novel low-frequency variants.

### 2.3 Comparison of association summary statistics with imputed GWAS

**Fig. 5.**
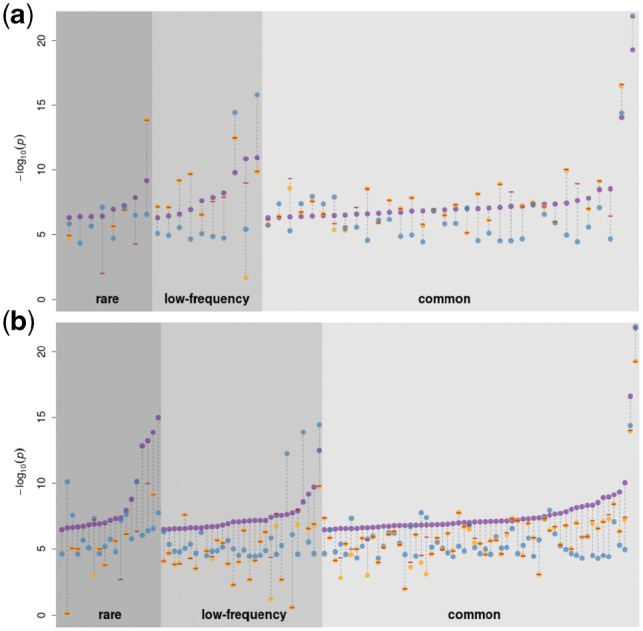
Association signals in the 1× WGS and imputed GWAS at *P*<5 × 10^−7^ for 57 quantitative traits in the 1225 samples with both imputed GWAS and low-depth WGS. Purple dots represent significant results in the imputed GWAS (**a**) and the 1× WGS (**b**) analysis. Orange dots, if present, denote the *P*-value of the same SNP in the other study. Blue dots represent the association *P*-value in a larger (*n*=1457) association study based on 22× WGS. Signals with a 22× WGS *P*-value above 5×10^−5^ were considered as false-positives in both studies and excluded from the plot. Red dashes indicate the minimum *P*-value among all tagging SNVs in the other dataset (*r*^2^>0.8). Absence of an orange dot and/or a red dash means that the variant was not present and/or no tagging variant could be found for that signal in the other study

1× WGS calls a larger number of variants and is noisier than imputed GWAS in the same samples. To evaluate how this difference affects association study power, we performed genome-wide association of 57 quantitative traits in 1225 overlapping samples with both imputed OmniExome and 1× WGS using both sources of genotype data. We then compared independent suggestively associated signals at *P* < 5×10^−7^ (Supplementary Table S2). These signals were then cross-referenced with a larger (*n* = 1457) study based on 22× WGS on the same traits in the same cohort (Gilly *et al.*, 2018). We only considered signals to be true if they displayed evidence for association with at most a two order of magnitude attenuation compared to our suggestive significance threshold (*P* < 5 ×10^−5^). According to this metric, 52 of 182 independent signals (28.5%) were true in the imputed GWAS, in contrast to 108 of 462 (23.4%) in the 1× study (Fig. 5). With an equal sample size and identically transformed traits, 1× therefore allowed to discover twice as many independent GWAS signals with almost identical truth sensitivity. Seven rare and three suggestive low-frequency variant associations in the 1× WGS data (9.2% of all signals) were driven by a variant not present and without a tagging SNP at *r*^2^ > 0.8 in the imputed GWAS, whereas the converse is true for only two rare variants in the imputed GWAS. Among variants called or tagged in the imputed GWAS, 4 rare, 11 low-frequency and 5 common SNV associations detected in the 1× (19% of total) are not seen associated below that threshold in the imputed GWAS. As expected, there are significantly fewer (3.8%, *P* = 0.01, one-sided chi-square proportion test) true associations in the imputed GWAS not recapitulated by the 1× study.

## 3 Discussion

In this work, we empirically demonstrate the relative merits of very low-depth WGS both in terms of variant discovery and association study power for complex quantitative traits compared to GWAS approaches. However, the advantages of 1× WGS have to be weighed against compute and financial cost considerations. As of summer 2018, 1× WGS on the HiSeq 4000 platform was approximately half of the cost of a dense GWAS array (e.g. Illumina Infinium Omni 2.5Exome-8 array), the same cost as a sparser chip such as the Illumina HumanCoreExome array, and half of the cost of WES at 50× depth. By comparison, 30× WGS was 23 or 15 times more costly depending on the sequencing platform (Illumina HiSeq 4000 or HiSeqX, respectively). The number of variants called by 1× WGS is lower than high-depth WGS, but is in the same order of magnitude, suggesting comparable disk storage requirements for variant calls. However, storage of the reads required an average 650 Mb per sample for CRAMs, and 1.3 Gb per sample for BAMs.

Genome-wide refinement and imputation of very low-depth WGS generates close to 50 times more variants than a GWAS chip. The complexity of the imputation and phasing algorithms used in this study is linear in the number of markers, linear in the number of target samples and quadratic in the number of reference samples (Browning and Browning, 2016), which results in a 50-fold increase in total processing time compared to an imputed GWAS study of equal sample size. In MANOLIS the genome was divided in 13 276 chunks containing equal number of SNVs, which took an average of 31 h each to refine and impute. The total processing time was 47 core-years (see Section 4 and Supplementary Fig. S2). This parallelization allowed processing the 1239 MANOLIS samples in under a month, and as imputation software continue to grow more efficient (Bycroft *et al.*, 2017), future pipelines should greatly simplify post-processing of very low-depth sequencing data.

As a proof of principle, we used imputed GWAS, 1 and 22× WGS in overlapping samples from an isolated population to assess how genotyping quality influences power in association studies. As we only wanted to study the implications of varying genotype qualities afforded by different designs on association *P*-values in a discovery setting, we considered only suggestively associated signals and did not seek replication in a larger cohorts for the discovered signals. In our study of 57 quantitative traits, we show that an 1×-based design allows the discovery of twice as many of the signals suggestively associated in the more accurate 22× WGS study, compared to the imputed GWAS design. Almost 10% of the suggestive signals arising in the 1× data are not discoverable in the imputed GWAS, but the great majority (96%) of imputed GWAS signals is found using the 1×.

The 1×-based study seems to discover more signals than the imputed GWAS across the MAF spectrum, and this remains true whether or not the signals are filtered for suggestive association *P*-value in the more accurate 22× based study (Supplementary Table S3). At first glance this suggests 1× WGS has better detection power than the imputed GWAS across the MAF spectrum, however it is unlikely that this is true for common variants, which are reliably imputed using chip data. Instead, this phenomenon may be due to a slightly less accurate imputation than in the GWAS dataset caused by a noisier raw genotype input (Supplementary Text). This effect is marginal, as evidenced by genome-wide concordance measures (Fig. 2) which are very high at the common end of the MAF spectrum. However, it is important to note that this slightly less accurate imputation can attenuate some signals as well as boosting others. For this reason, we would recommend relaxing the discovery significance threshold in 1× studies in order to capture those less well imputed, signal-harboring variants, followed by rigorous replication in larger cohorts and direct validation of genotypes.

Our study’s intent was to focus on the performance on commonly used general-purpose tools for low-depth sequencing data in isolates, both for genotype calling (GATK) and imputation (BEAGLE, IMPUTE). There are ongoing efforts to leverage the specificities of both low-depth sequencing (Davies *et al.*, 2016) (https://www.gencove.com) and of isolated populations (Livne *et al.*, 2015). The popularity and long-term support of established generic methods is an advantage when running complex study designs, as has been shown in other isolate studies (Herzig *et al.*, 2018). This study focused on SNVs, excluding INDELS due to high expected noise levels, poor call rate and genotype accuracy, as well as lower imputation quality using general-purpose tools. Very low-depth-based studies rely heavily on imputation to complete the sparse picture of variation painted by few and far between reads. The accurate detection and genotyping of larger variants such as structural variants, which relies more heavily on read-based evidence such as split reads, insert size or depth anomalies, and which are currently poorly imputed, is therefore likely to remain a methodological challenge for low-depth WGS studies.

We show that very low-depth WGS allows the accurate assessment of most common and low-frequency variants captured by imputed GWAS designs and achieves denser coverage of the low-frequency and rare end of the allelic spectrum, albeit at an increased computational cost. This allows very low-depth sequencing studies to recapitulate signals discovered by imputed chip-based efforts, and to discover significantly associated variants missed by GWAS imputation (Gilly *et al.*, 2016). Although cohort-wide high-depth WGS remains the gold standard for the study of rare and low-frequency variation, very low-depth WGS designs using population-specific haplotypes for imputation remain a viable alternative when studying populations poorly represented in existing large reference panels.

## 4 Materials and methods

### 4.1 Cohort details

The HELIC (www.helic.org) MANOLIS (Minoan Isolates) collection focuses on Anogia and surrounding Mylopotamos villages on the Greek island of Crete. All individuals were required to have at least one parent from the Mylopotamos area to enter the study. Recruitment was primarily carried out at the village medical centers. The study includes biological sample collection for DNA extraction and lab-based blood measurements, and interview-based questionnaire filling. The phenotypes collected include anthropometric and biometric measurements, clinical evaluation data, biochemical and hematological profiles, self-reported medical history, demographic, socioeconomic and lifestyle information. The study was approved by the Harokopio University Bioethics Committee and informed consent was obtained from every participant.

### 4.2 Sequencing

Sequencing and mapping for the 990 MANOLIS samples at 1× depth has been described previously (Gilly *et al.*, 2016), as well as for 249 MANOLIS samples at 4× (Southam *et al.*, 2017), and for 1457 samples at 22× (Gilly *et al.*, 2018). For comparison, five samples from the cohort were also whole-exome sequenced at an average depth of 75×. We use a standard read alignment and variant calling pipeline using samtools (Li *et al.*, 2009) and GATK (McKenna *et al.*, 2010), which is described in detail in the Supplementary Text. A Venn diagram detailing the overlap between the sequenced and genotyped datasets is given in Supplementary Figure S3.

### 4.3 Variant filtering

Variant quality score recalibration was performed using GATK VQSR v.3.1.1. However, using the default parameters for the VQSR mixture model yields poor filtering, with a Ti/Tv ratio dropoff at 83% percent sensitivity and a transition/transversion (Ti/Tv) ratio of 1.8 for high-quality tranches (Supplementary Fig. S4a). We therefore ran exploratory runs of VQSR across a range of values for the model parameters, using the dropoff point of the Ti/Tv ratio below 2.0 as an indicator of good fit (Supplementary Fig. S5). A small number of configurations outperformed all others, which allowed us to select an optimal set of parameters. For the chosen set of parameters, false-positive rate is estimated at 10 ± 5% (Supplementary Fig. S4b). Indels were excluded from the dataset out of concerns for genotype quality. We found that the version of VQSR, as well as the annotations used to train the model, had a strong influence on the quality of the recalibration (Supplementary Fig. S5 and Supplementary Text).

### 4.4 Comparison with downsampled whole genomes

For quality control purposes, reads from 17 of the well-characterized Platinum Genomes sequenced by Illumina at 50× depth (Eberle *et al.*, 2017), and downsampled to 1× depth using samtools (Li *et al.*, 2009) were included in the merged BAM file. VQSR-filtered calls were then compared to the high-confidence call sets made available by Illumina for those samples. A total of 524 331 out of the 4 348 092 non-monomorphic variant sites were not present in the high-confidence calls, whereas 1 246 403 of the 5 070 164 non-monomorphic high-confidence Platinum variants were not recapitulated in the 1× data. This corresponds to an estimated false-positive rate of 12% and false-negative rate of 24.6%. Both unique sets had a much higher proportion of singletons (corresponding to MAF < 2.9%) than the entire sets (57.9 versus 19.9% of singletons among 1× calls and 51 versuss 18.1% among high-confidence calls), which suggests that a large fraction of the erroneous sites lies in the low-frequency and rare part of the allelic spectrum. However, genotype accuracy is poor, to the point where it obscures peculiarities in the distribution of allele counts (Supplementary Fig. S6). Due to these being present in the 1000 genomes reference panel, we remove the 17 Platinum Genomes prior to imputation.

### 4.5 Genotype refinement and imputation

#### Evaluation of pipelines

The authors of SHAPEIT (Delaneau *et al.*, 2013) advise to phase whole chromosome when performing pre-phasing in order to preserve downstream imputation quality. This approach is computationally intractable for the 1× datasets, where the smallest chromosomes contain almost seven times more variants than the largest chromosomes in a GWAS dataset.

For benchmarking purposes, we designed 13 genotype refinement pipelines involving Beagle v4.0 (Browning and Browning, 2007) and IMPUTE2 (Howie *et al.*, 2009, 2011) using a 1000 Genomes phase 1 reference panel, which we evaluated against minor allele concordance. All pipelines were run using the vr-runner scripts (https://github.com/VertebrateResequencing/vr-runner). Pipelines involving Beagle with the use of a reference panel ranked consistently better (Supplementary Fig. S1), with a single run of reference-based refinement using Beagle outperforming all other runs. IMPUTE2 performed worst on its own, whether with or without reference panel; in fact the addition of a reference panel did not improve genotype quality massively. Phasing with Beagle without an imputation panel improved genotype quality, before or after IMPUTE2.

Halving the number of SNVs per refinement chunk (including 500 flanking positions) from the 4000 recommended by the vr pipelines resulted in only a modest loss of genotype quality in the rare part of the allelic spectrum (Supplementary Fig. S7), while allowing for a 2-fold increase in refinement speed. Genotype quality dropped noticeably for rare variants when imputation was turned on (Supplementary Fig. S7), but remained high for low-frequency and common ones. A reference-free run of Beagle allowed to phase all positions and remove genotype missingness with no major impact on quality and a low computational cost. We initially also tested thunderVCF (https://genome.sph.umich.edu/wiki/ThunderVCF) for phasing sites, however, the program took more than 2 days to run on 5000 SNV chunks and was abandoned.

#### Production pipeline for the MANOLIS cohort

The pipeline with best minor allele concordance across the board used Beagle v.4 (Browning and Browning, 2007) to perform a first round of imputation-based genotype refinement on 1239 HELIC-MANOLIS variant call sets, using the aforementioned reference panel. This was followed by a second round of reference-free imputation, using the same software. For production, we used a previously-described (Southam *et al.*, 2017) reference panel composed of 10 244 haplotypes from the 1000 Genomes Project Phase 1 (*n* = 1092), 249 MANOLIS samples sequenced at 4× depth, as well as the two cohorts included in the UK10K study (UK10K Consortium *et al.*, 2015) [TwinsUK (Moayyeri *et al.*, 2013) and ALSPAC (Golding *et al.*, 2001) (total *n* = 3781, 7× WGS)]. Alleles in the reference panel were matched to the reference allele in the called dataset. Positions where the alleles differed between the called and reference datasets were removed from both sources. Indels were filtered out due to poor calling quality.

#### Variant-level QC

Beagle provides two position level imputation metrics, allelic R-squared and dosage R-squared. Both measures are highly correlated (Supplementary Fig. S8a). Values between 0.3 and 0.8 are typically used for filtering (Brian Browning, personal communication). In the 1× dataset 59% and 91% of imputed variants lie below those two thresholds, respectively. The distribution of scores does not provide an obvious filtering threshold (Supplementary Fig. S8b) due to its concavity. Since most imputed variants are rare and R-squared measures are highly correlated with MAF, filtering by allelic R-squared and dosage R-squared would be similar to imposing a MAF threshold (Supplementary Fig. S8c and d). Moreover, due to a technical limitation of the vr-runner pipelines, imputation quality measures were not available for refined positions at the time of analysis, only imputed ones. Therefore, we did not apply any imputation quality filter in downstream analyses.

### 4.6 Sample QC

Due to the sparseness of the 1× datasets, sample-level QC was performed after imputation. A total of five samples were excluded from the MANOLIS 1× cohort following PCA-based ethnicity checks.

### 4.7 Comparison with WES

A set of high-confidence genotypes was generated for the five exomes in MANOLIS using filters for variant quality (QUAL > 200), call rate (AN = 10, 100%) and depth (250×). These filters were derived from the respective distributions of quality metrics (Supplementary Fig. S9).

When compared to five whole-exome sequences from the MANOLIS cohort, imputed 1× calls recapitulated 77.2% of non-monomorphic, high-quality exome sequencing calls. Concordance was high, with only 3.5% of the overlapping positions exhibiting some form of allelic mismatch. When restricting the analysis to singletons, 9105 (58%) of the 15 626 high-quality singletons in the exomes were captured, with 21% of the captured positions exhibiting false-positive genotypes (AC > 1). To assess false-positive call rate, we extracted 1× variants falling within the 71 627 regions targeted by the Agilent design file for WES in overlapping samples, and compared them to those present in the unfiltered WES dataset. A total of 103 717 variants were called in these regions from WES sequences, compared to 58 666 non-monomorphic positions in the 1× calls and 1419 (2.4%) of these positions were unique to the 1× dataset, indicating a low false-positive rate in exonic regions post-imputation.

### 4.8 Genetic relatedness matrix

Relatedness was present at high levels in our cohort, with 99.5% of samples having at least one close relative (estimated $\hat{\pi }>0.1$) and an average number of close relatives of 7.8. In order to correct for this close kinship typical of isolated cohorts, we calculated a genetic relatedness matrix using GEMMA (Zhou and Stephens, 2012). Given the isolated nature of the population and the specificities of the sequencing dataset, we used different variant sets to calculate kinship coefficients. Using the unfiltered 1× variant dataset produced the lowest coefficients (Supplementary Fig. S10a), whereas well-behaved set of common SNVs (Arthur *et al.*, 2017) produced the highest, with an average difference of 3.67×10^−3^. Filtering for MAF increased the inferred kinship coefficients. Generally, the more a variant set was sparse and enriched in common variants, the higher the coefficients were. However, these differences only had a marginal impact on association statistics, as evidenced by a lambda median statistic difference of 0.02 between the two most extreme estimates of relatedness when used for a genome-wide association of triglycerides (Supplementary Fig. S10b). For our association study, we used LD-pruned 1× variants filtered for MAF < 1% and Hardy Weinberg equilibrium *P* < 1 ×10^−5^ to calculate the relatedness matrix, which translated into 2 848 245 variants for MANOLIS.

### 4.9 Single-point association

#### Pipeline

For association, 57 phenotypes were prepared, with full details of the trait transformation, filters and exclusions described in Supplementary Table S4. The ‘transformPhenotype’ (https://github.com/wtsi-team144/transformPhenotype) R script was used to apply a standardized preparation for all phenotypes. Association analysis was performed using the linear mixed model implemented in GEMMA (Zhou and Stephens, 2012) on all variants with minor allele count >2 (14 948 665 out of 30 483 158 variants in MANOLIS). Singletons and doubletons are removed due to overall low minor allele concordance. We used the aforementioned centered kinship matrix. GC-corrected *P*-values from the score test (p_score) were used due to residual inflation being present in some traits. Signals were extracted using the peakplotter software (https://github.com/wtsi-team144/peakplotter) using a window size of 1 Mb.

## Supplementary Material

bty1032_Supplementary_Data

## References

[bty1032-B1] 1000 Genomes Project Consortium et al (2015) A global reference for human genetic variation. Nature, 526, 68–74. 26432245 10.1038/nature15393 PMC4750478

[bty1032-B2] Alex Buerkle C., Gompert Z. (2013) Population genomics based on low coverage sequencing: how low should we go? Mol. Ecol., 22, 3028–3035. 23174005 10.1111/mec.12105

[bty1032-B3] Arthur R. et al (2017) AKT: ancestry and kinship toolkit. Bioinformatics, 33, 142–144. 27634946 10.1093/bioinformatics/btw576

[bty1032-B4] Astle W.J. et al (2016) The allelic landscape of human blood cell trait variation and links to common complex disease. Cell, 167, 1415–1429. 27863252 10.1016/j.cell.2016.10.042 PMC5300907

[bty1032-B5] Brody J.A. et al (2017) Analysis commons, a team approach to discovery in a big-data environment for genetic epidemiology. Nat. Genet., 49, 1560–1563. 29074945 10.1038/ng.3968 PMC5720686

[bty1032-B6] Browning B.L., Browning S.R. (2016) Genotype Imputation with Millions of Reference Samples. Am. J. Hum. Genet., 98, 116–126. 26748515 10.1016/j.ajhg.2015.11.020 PMC4716681

[bty1032-B7] Browning S.R., Browning B.L. (2007) Rapid and accurate haplotype phasing and missing-data inference for whole-genome association studies by use of localized haplotype clustering. Am. J. Hum. Genet., 81, 1084–1097. 17924348 10.1086/521987 PMC2265661

[bty1032-B8] Bycroft C. et al (2017) Genome-wide genetic data on ∼500, 000 UK Biobank participants. https://www.ncbi.nlm.nih.gov/pubmed/?term=30305743.

[bty1032-B9] Converge Consortium. (2015) Sparse whole-genome sequencing identifies two loci for major depressive disorder. Nature, 523, 588–591. 26176920 10.1038/nature14659 PMC4522619

[bty1032-B10] Davies R.W. et al (2016) Rapid genotype imputation from sequence without reference panels. Nat. Genet., 48, 965–969. 27376236 10.1038/ng.3594 PMC4966640

[bty1032-B11] Delaneau O. et al (2013) Haplotype estimation using sequencing reads. Am. J. Hum. Genet., 93, 687–696. 24094745 10.1016/j.ajhg.2013.09.002 PMC3791270

[bty1032-B12] Eberle M.A. et al (2017) A reference data set of 5.4 million phased human variants validated by genetic inheritance from sequencing a three-generation 17-member pedigree. Genome Res., 27, 157–164. 27903644 10.1101/gr.210500.116 PMC5204340

[bty1032-B13] Gilly A. et al (2016) Very low-depth sequencing in a founder population identifies a cardioprotective APOC3 signal missed by genome-wide imputation. Hum. Mol. Genet., 25, 2360–2365. 27146844 10.1093/hmg/ddw088 PMC5081052

[bty1032-B14] Gilly A. et al (2018) Cohort-wide deep whole genome sequencing and the allelic architecture of complex traits. Nat. Commun., 9, 4674. 30405126 10.1038/s41467-018-07070-8 PMC6220258

[bty1032-B16] Golding J. et al (2001) ALSPAC–the Avon Longitudinal Study of Parents and Children. I. Study methodology. Paediatr. Perinat. Epidemiol., 15, 74–87. 11237119 10.1046/j.1365-3016.2001.00325.x

[bty1032-B17] Herzig A.F. et al (2018) Strategies for phasing and imputation in a population isolate. Genet. Epidemiol., 42, 201–213. 29319195 10.1002/gepi.22109

[bty1032-B18] Howie B. et al (2011) Genotype imputation with thousands of genomes. G3 (Bethesda), 1, 457–470. 22384356 10.1534/g3.111.001198 PMC3276165

[bty1032-B19] Howie B.N. et al (2009) A flexible and accurate genotype imputation method for the next generation of genome-wide association studies. PLoS Genet., 5, e1000529. 19543373 10.1371/journal.pgen.1000529 PMC2689936

[bty1032-B20] Le S.Q., Durbin R. (2011) SNP detection and genotyping from low-coverage sequencing data on multiple diploid samples. Genome Res., 21, 952–960. 20980557 10.1101/gr.113084.110 PMC3106328

[bty1032-B21] Li H. et al (2009) The sequence alignment/map format and SAMtools. Bioinformatics, 25, 2078–2079. 19505943 10.1093/bioinformatics/btp352 PMC2723002

[bty1032-B22] Livne O.E. et al (2015) PRIMAL: fast and accurate pedigree-based imputation from sequence data in a founder population. PLoS Comput. Biol., 11, e1004139. 25735005 10.1371/journal.pcbi.1004139 PMC4348507

[bty1032-B23] McCarthy S. et al (2016) A reference panel of 64, 976 haplotypes for genotype imputation. Nat. Genet., 48, 1279–1283. 27548312 10.1038/ng.3643 PMC5388176

[bty1032-B24] McKenna A. et al (2010) The Genome Analysis Toolkit: a MapReduce framework for analyzing next-generation DNA sequencing data. Genome Res., 20, 1297–1303. 20644199 10.1101/gr.107524.110 PMC2928508

[bty1032-B25] Moayyeri A. et al (2013) The UK Adult Twin Registry (TwinsUK Resource). Twin Res. Hum. Genet., 16, 144–149. 23088889 10.1017/thg.2012.89 PMC3927054

[bty1032-B26] Pasaniuc B. et al (2012) Extremely low-coverage sequencing and imputation increases power for genome-wide association studies. Nat. Genet., 44, 631–635. 22610117 10.1038/ng.2283 PMC3400344

[bty1032-B27] Southam L. et al (2017) Whole genome sequencing and imputation in isolated populations identify genetic associations with medically-relevant complex traits. Nat. Commun., 8, 15606. 28548082 10.1038/ncomms15606 PMC5458552

[bty1032-B29] Tachmazidou I. et al (2017) Whole-genome sequencing coupled to imputation discovers genetic signals for anthropometric traits. Am. J. Hum. Genet., 100, 865–884. 28552196 10.1016/j.ajhg.2017.04.014 PMC5473732

[bty1032-B30] UK10K Consortium. et al (2015) The UK10K project identifies rare variants in health and disease. Nature, 526, 82–90. 26367797 10.1038/nature14962 PMC4773891

[bty1032-B31] Zhou X., Stephens M. (2012) Genome-wide efficient mixed-model analysis for association studies. Nat. Genet., 44, 821–824. 22706312 10.1038/ng.2310 PMC3386377

