# Supplementary material for: Very low-depth whole-genome sequencing in complex trait association studies
Source: Bioinformatics. 2018 Dec 21;35(15):2555–61. doi: 10.1093/bioinformatics/bty1032 (PMC6662288; doi:10.1093/bioinformatics/bty1032)
Supplement: bty1032_Supplementary_Data [file bty1032_supplementary_data.zip › bty1032-suppl_data/Gilly.Revision.Supplementary.docx]

**Supplementary Data**

**Supplementary Figure 1: Minor allele concordance for genotype refinement pipelines**. Chromosome 11 data, no imputation was performed, only positions called by sequencing are evaluated. Missingness is zero for positions included in the reference panel when a panel is present, and zero across all positions when performing reference-free genotype refinement. Pipelines using Beagle v4 with a reference panel are drawn in blue, pipelines not involving Beagle or not using a reference panel are coloured red. 1KG=1000 Genomes phase I reference panel. Details of the pipelines are presented in Supplementary Table 1.


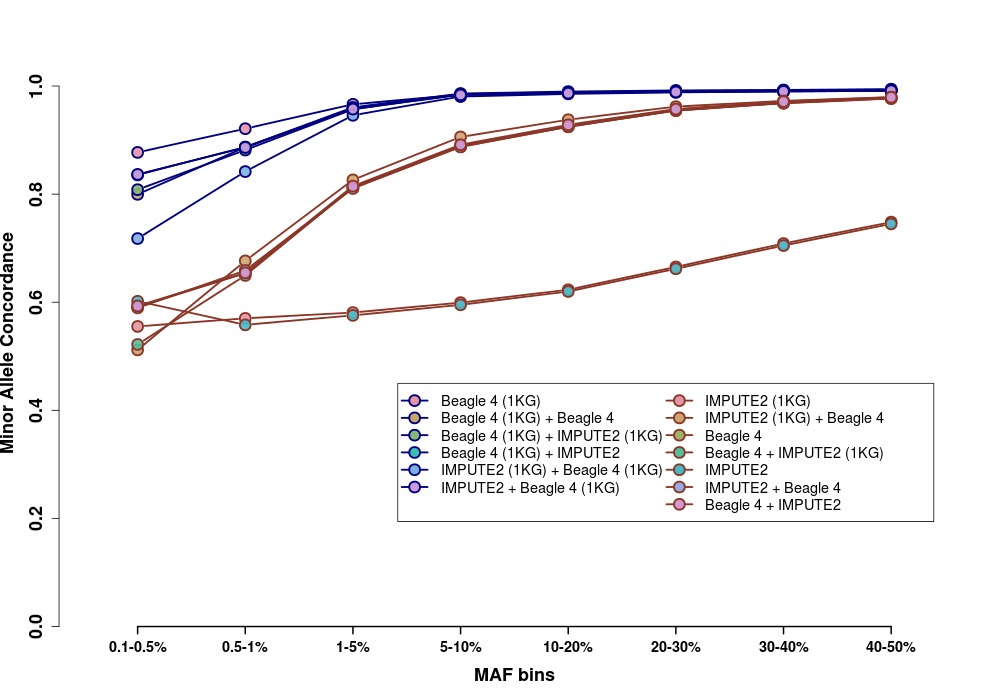


**Supplementary Figure 2: Genotype refinement and imputation compute time, measured in core hours, on chunks of 2,000 SNV genome-wide for the MANOLIS cohort**. Some chunks located in regions such as the major histocompatibility complex took up to 271 hours (11 days) to complete.

**
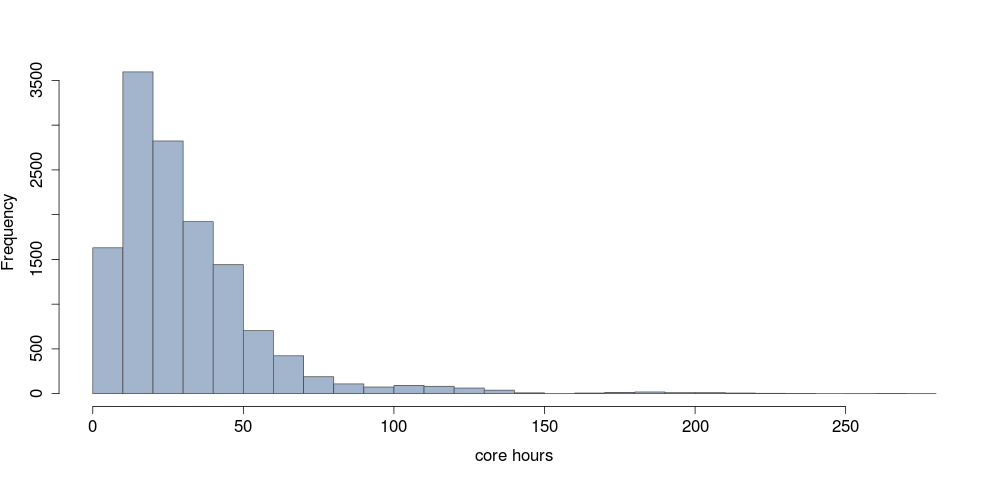
**

**Supplementary Figure 3: Venn diagram of the sequenced and genotyped datasets in MANOLIS.** Figure generated using <http://bioinformatics.psb.ugent.be/webtools/Venn/>


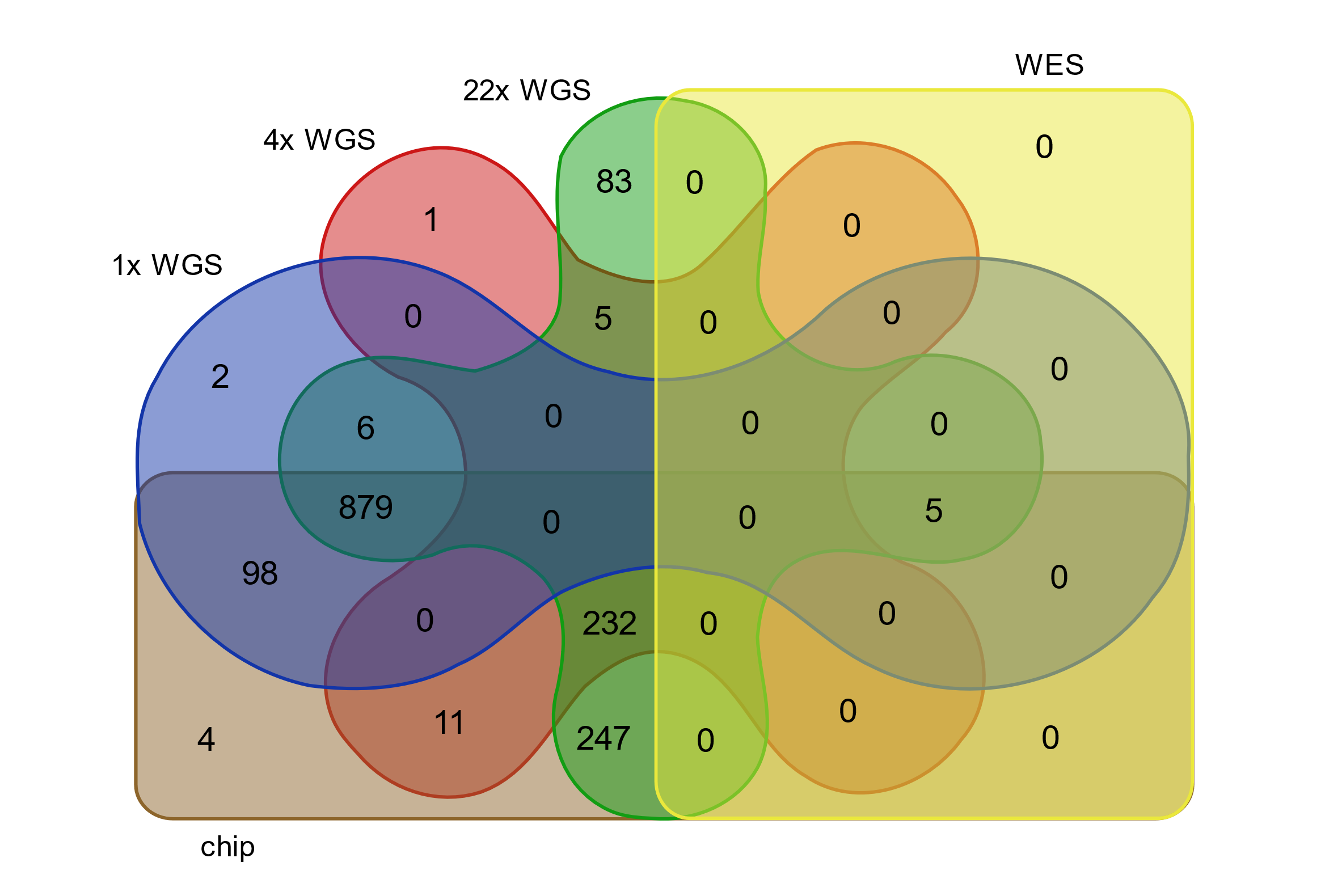


**Supplementary Figure 4: VQSR recalibration curves** for the MANOLIS cohort. **a.** VQSR version 3.1.1. with default parameters, **b.** using the optimal set of parameters. Ti/Tv ratio for novel variants is given by VQSR. The fraction of true sites called is the proportion of variants in the positive training set called at that tranche or below. The estimated false positive rate is calculated as $FP=\frac{TiTv_{expected}-TiTv_{novel}}{TiTv_{expected}-TiTv_{FP}}$ where $TiTv_{FP}=0.5$ and $TiTv_{expected}$ is 2.6 for the lower dashed curve, 2.1 for the upper dashed curve, and 2.3 for the solid curve.


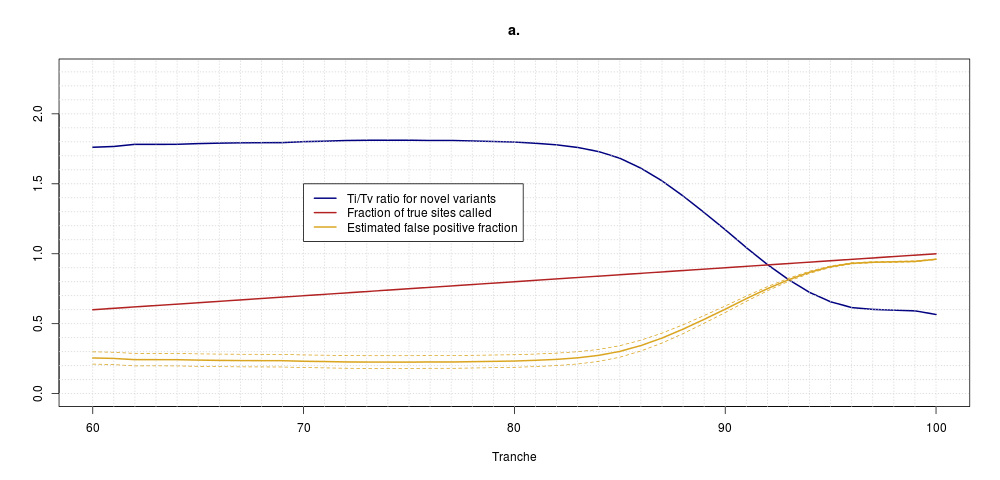


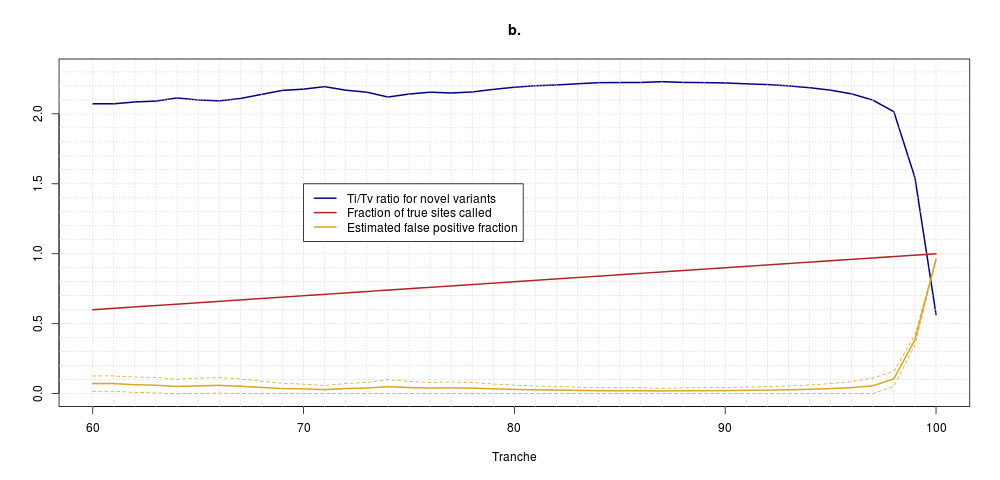


**Supplementary Figure 5: VQSR optimisation grid for two versions of VQSR**. a. version 2.7.2, b. version 3.1.1. The bottom axis represents the minimum number of variants in the negative training set (minNumBadVariants), the vertical axis is the quality field threshold (qualThreshold) and the top axis is the maximum number of Gaussian distributions in the mixture model (maxGaussians).


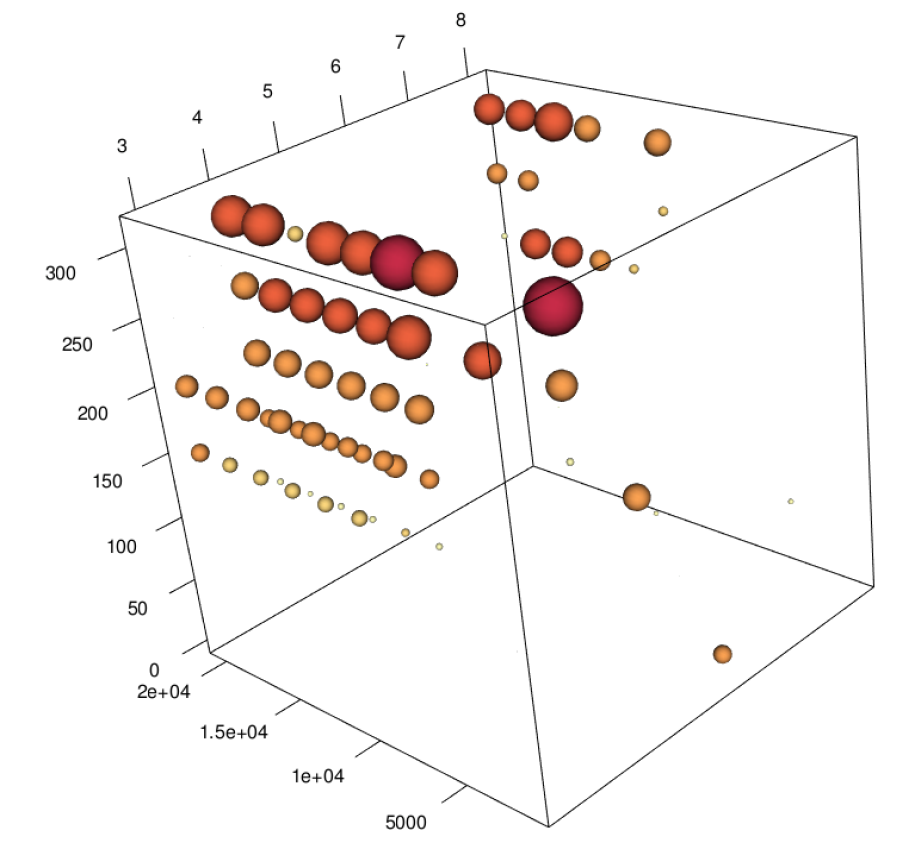
**a.**

20000

15000

10000


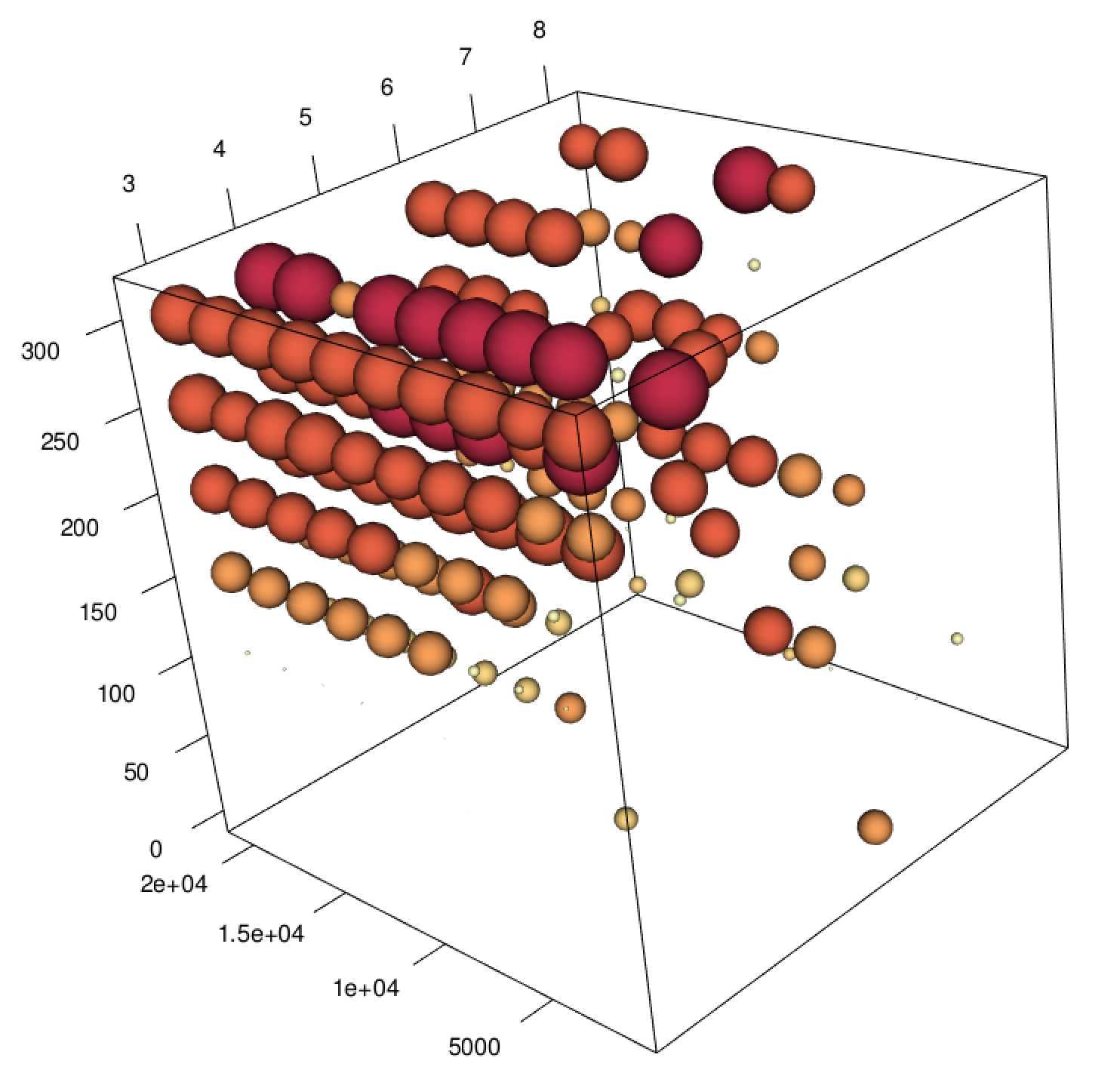


5000

**b.**

20000

15000

10000

5000

**Supplementary**
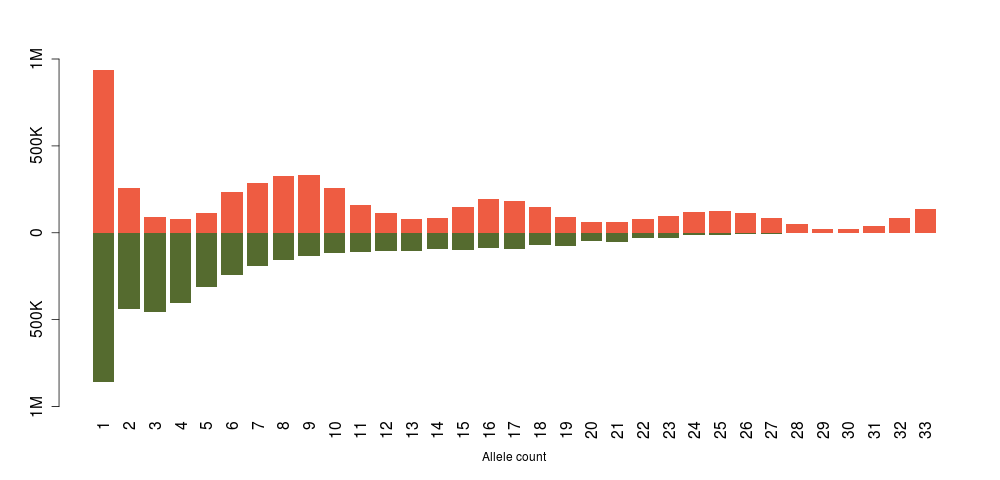
**Figure 6: Allelic spectra for non-monomorphic sites in the Platinum Genomes samples.** Orange bars are the high-confidence calls from high-depth WGS, green bars are calls from downsampled BAMs at 1x. Differences in the shape of the distribution are likely due to the scarcity of information provided by the 1x data, which does not allow to recapitulate the family structure in the CEPH/Utah pedigree 1463, which composes the Platinum Genomes.

**Supplementary**
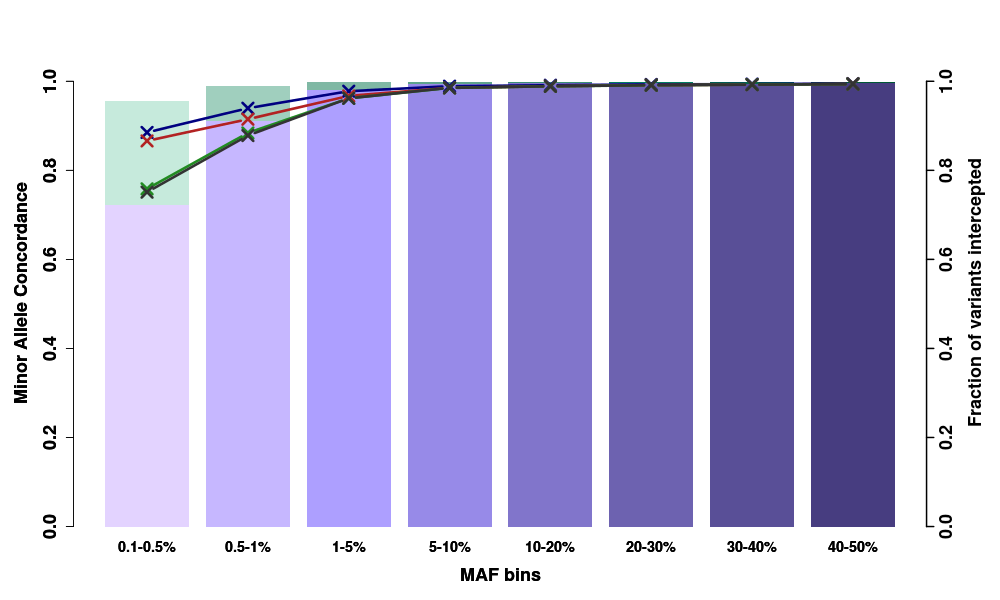
**Figure 7: Effect of decreasing chunk size and adding imputed variants on minor allele concordance and variant overlap.** Concordance and overlap are evaluated against GWAS data. Purple bars: variant overlap in the refined-only dataset. Green bars: Imputed variants. Blue curve: Minor allele concordance, refined variants only using the 3-way reference panel, large chunk size (3,000 variants, 1000 flanking variants). Red curve: Refined variants only using the 3-way reference panel, small chunk size (1,500 variants, 500 flanking variants). Green curve: Imputed variants added, small chunk size. Black curve: Beagle phasing run added (no missingness).

**Supplementary**
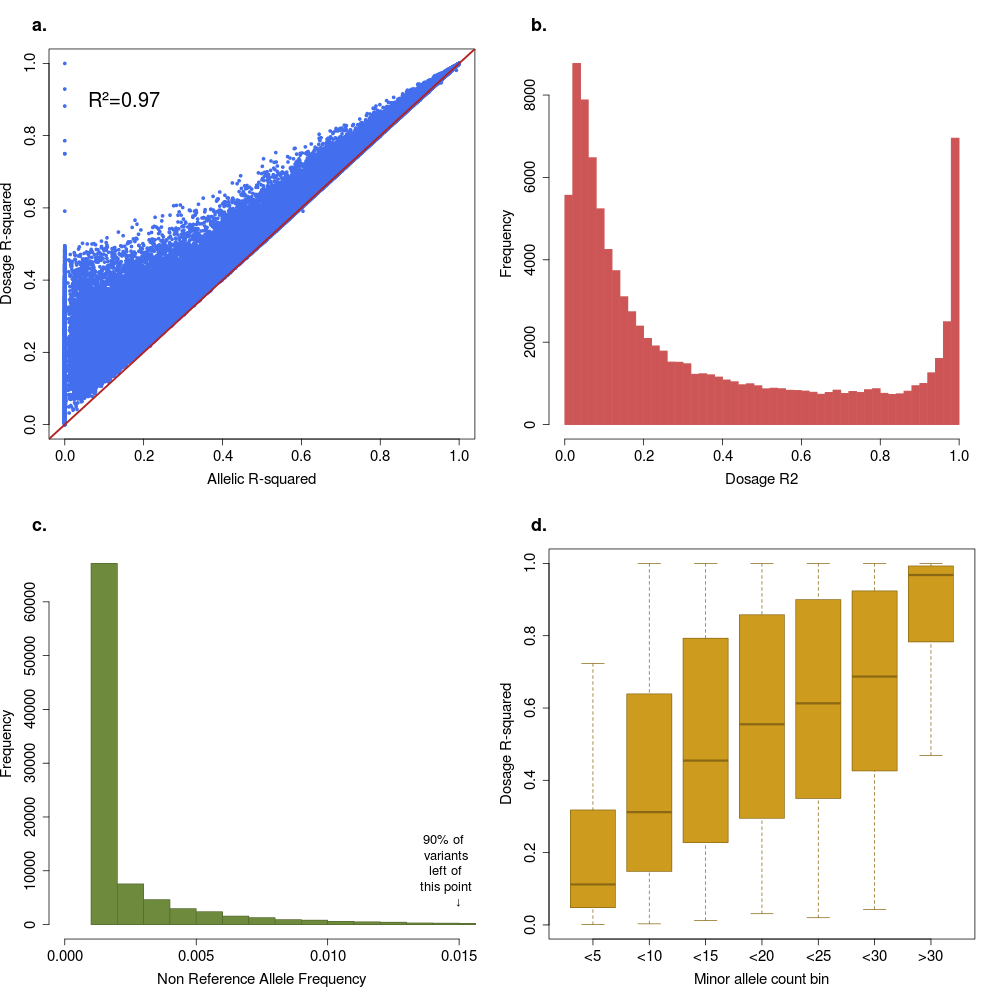
**Figure 8: Distributions and relationship between the two imputation accuracy measures provided by Beagle**. Chromosome 11 data, imputed positions only. **a.** Correlation of Beagle imputation quality metrics. **b.** Distribution of Dosage R-squared. **c.** Allele frequencies of imputed variants. **d.** Dosage R-squared and allele frequency.

**Supplementary Figure 9: Quality metrics for the 5 MANOLIS WES sequences.** Metrics were extracted for chromosome 11. a. Variant quality score distribution. b. Call rate (AN) per quality score. c. Cumulative depth (DP) per quality score. d. Depth per sample. e. Quality per called alternative allele count (AC), on call-rate 100% SNVs only. Chosen thresholds are displayed in red where applicable.


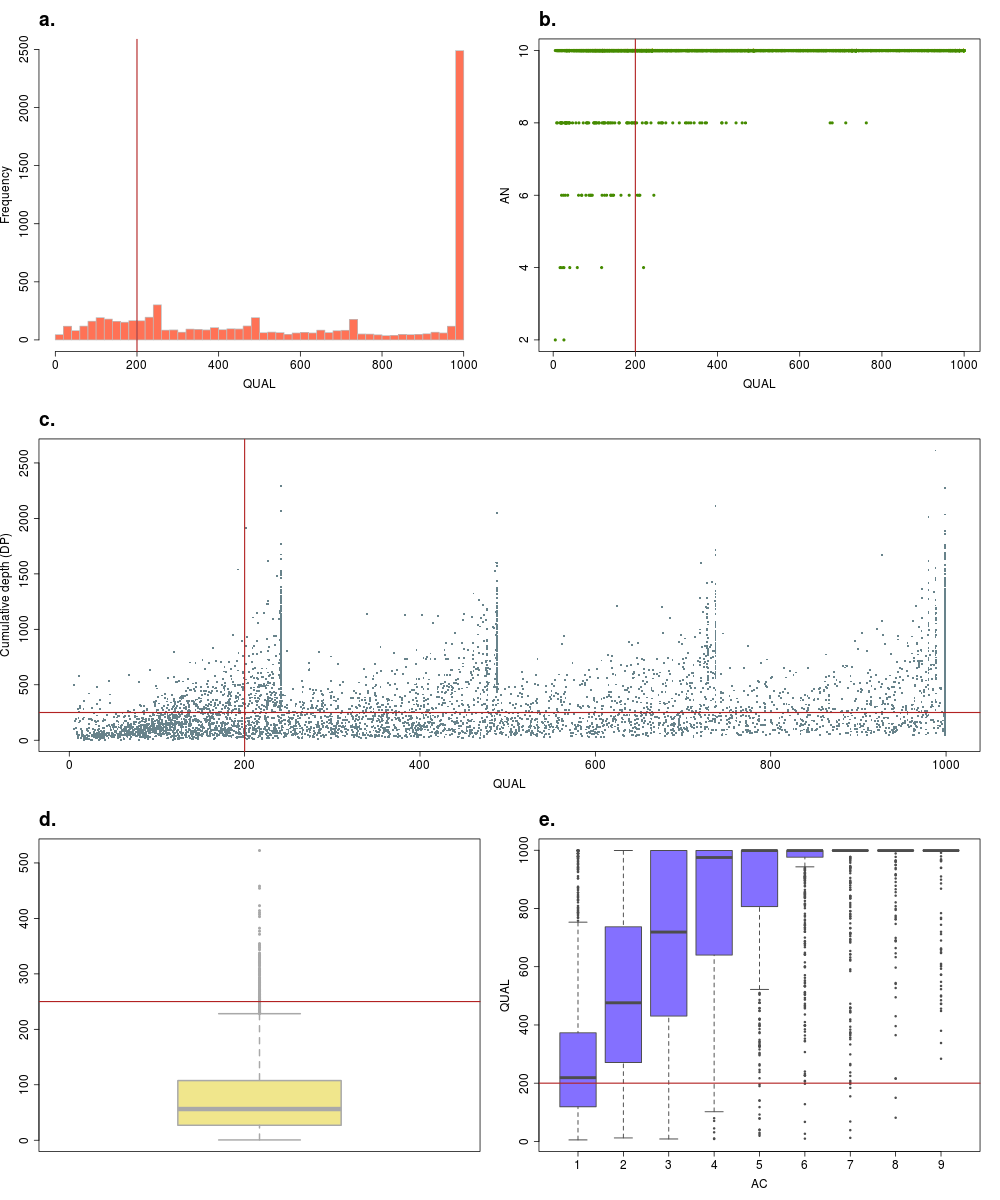


**
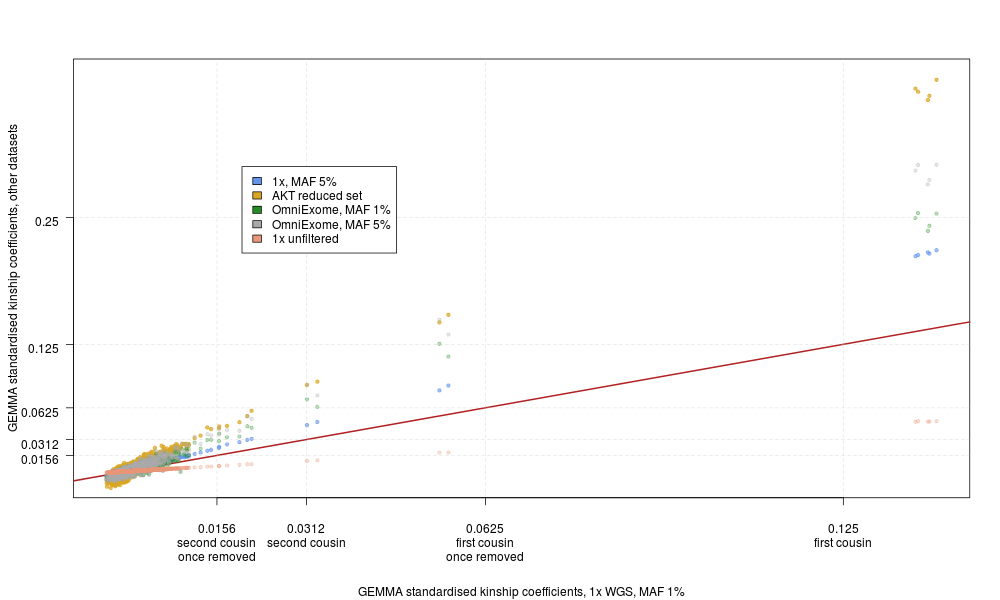
Supplementary Figure 10: Kinship coefficients have a limited effect on association statistics. a.** Kinship coefficients in the genetic relatedness matrix are calculated using GEMMA using genome-wide data across 5 sets of sites, and are compared to the coefficients used in the analysis (red line, 20,667,092 sites, 1x WGS data, MAF>1%). All datasets are LD-pruned and filtered for Hardy-Weinberg equilibrium p<1x10^-5^. **b.** QQ-plots of a triglycerides (TG) association using the highest (left) and lowest (right) kinship coefficients in **a**. The difference in lambda inflation factors is small (0.0172).

a.

| 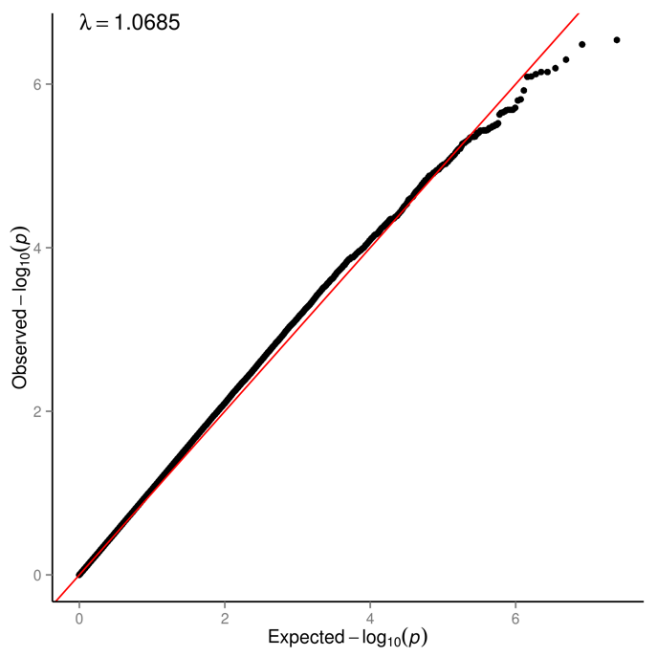b. | 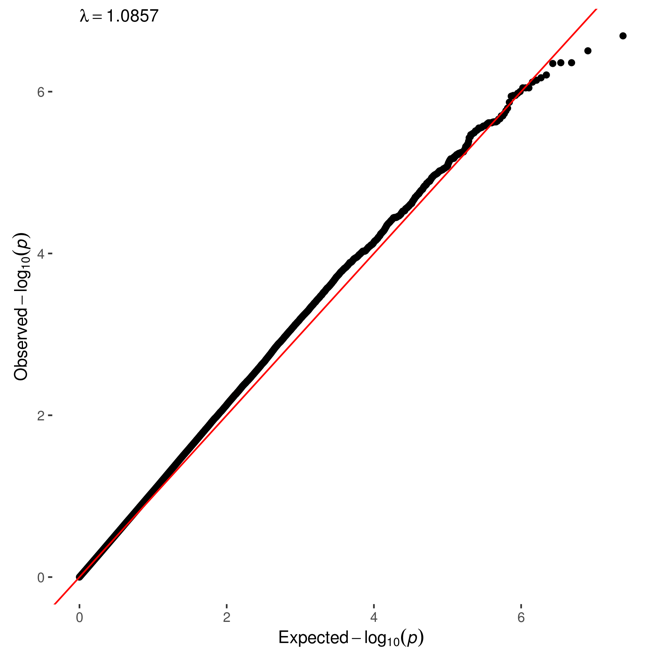 |
| --- | --- |

**Supplementary Tables**

**Supplementary Table 1: Detail of the benchmarked genotype refinement and imputation pipelines.** Dashes indicate that no second step was performed.

| **Name** | **First pass** | | **Second pass** | |
| --- | --- | --- | --- | --- |
|  | **Software** | **Reference Panel** | **Software** | **Reference Panel** |
| Beagle 4 (1KG) | Beagle v.4 | 1000 Genomes Phase 1 | - | - |
| Beagle 4 (1KG) + Beagle 4 | Beagle v.4 | 1000 Genomes Phase 1 | Beagle v.4 | None - phasing mode |
| Beagle 4 (1KG) + IMPUTE2 (1KG) | Beagle v.4 | 1000 Genomes Phase 1 | IMPUTE V2 | 1000 Genomes Phase 1 |
| Beagle 4 (1KG) + IMPUTE2 | Beagle v.4 | 1000 Genomes Phase 1 | IMPUTE V2 | None – phasing mode |
| IMPUTE2 (1KG) + Beagle 4 (1KG) | IMPUTE V2 | 1000 Genomes Phase 1 | Beagle v.4 | 1000 Genomes Phase 1 |
| IMPUTE2 + Beagle 4 (1KG) | IMPUTE V2 | None - phasing mode | Beagle v.4 | 1000 Genomes Phase 1 |
| IMPUTE2 (1KG) | IMPUTE V2 | 1000 Genomes Phase 1 | - | - |
| IMPUTE2 (1KG) + Beagle 4 | IMPUTE V2 | 1000 Genomes Phase 1 | Beagle v.4 | None – phasing mode |
| Beagle 4 | Beagle v.4 | None – phasing mode | - | - |
| Beagle 4 + IMPUTE2 (1KG) | Beagle v.4 | None – phasing mode | IMPUTE V2 | 1000 Genomes Phase 1 |
| IMPUTE2 | IMPUTE V2 | None – phasing mode | - | - |
| IMPUTE2 + Beagle 4 | IMPUTE V2 | None – phasing mode | Beagle v.4 | None – phasing mode |
| Beagle 4 + IMPUTE2 | Beagle v.4 | None – phasing mode | IMPUTE V2 | None – phasing mode |

**Supplementary Table 2: Lead variants in signals arising in the imputed GWAS (a) and the 1x WGS studies (b).** Trait summaries and transformations are given in Supplementary Table 3. The effect size (beta) standard error (se), Wald, Likelihood Ratio and Score test values are from the GEMMA output. Variants are annotated using Ensembl via the REST API. Most severe consequence, rsID and mapped gene are obtained through Variant Effect Predictor. Previous associations are obtained through the Ensembl phenotype annotation overlap.

**a. Imputed GWAS**

| **trait** | **chr** | **ps** | **minor** | **major** | **MAF** | **beta** | **se** | **Wald test p-value** | **LR test p-value** | **score test p-value** | **previous associations** | **ensembl most severe consequence** | **rsID if applicable** | **mapped gene if applicable** |
| --- | --- | --- | --- | --- | --- | --- | --- | --- | --- | --- | --- | --- | --- | --- |
| LDL | 2 | 22695702 | A | G | 0.008 | 1.21 | 0.24 | 4.90E-07 | 4.76E-07 | 6.35E-07 | none | intron_variant | rs182210478 | AC068490.2;AC096570.2 |
| BGP | 4 | 78130533 | T | C | 0.002 | -2.58 | 0.51 | 4.05E-07 | 4.12E-07 | 5.92E-07 | none | intron_variant | 4:78130533 | CCNG2 |
| Fe_iron | 2 | 182072184 | C | CT | 0.007 | 1.30 | 0.26 | 4.14E-07 | 4.04E-07 | 5.48E-07 | none | intron_variant | chr2:182072184 | AC104820.2 |
| MCV | 11 | 10720033 | G | T | 0.004 | -1.95 | 0.38 | 3.11E-07 | 3.76E-07 | 7.36E-07 | none | upstream_gene_variant | chr11:10720033 |  |
| RG | 15 | 89987286 | G | A | 0.003 | -2.16 | 0.408 | 1.07E-07 | 1.12E-07 | 1.69E-07 | none | intergenic_variant | 15:89987286 |  |
| RG | 8 | 18632326 | T | C | 0.004 | -1.97 | 0.36 | 5.80E-08 | 5.76E-08 | 8.73E-08 | none | non_coding_transcript_exon_variant | rs76185967 | PSD3;RPL35P6 |
| RDW | 11 | 10720033 | G | T | 0.006 | -1.83 | 0.32 | 1.38E-08 | 1.35E-08 | 2.42E-08 | none | upstream_gene_variant | chr11:10720033 |  |
| HDL | 16 | 73298565 | C | T | 0.01 | -1.37 | 0.22 | 4.91E-10 | 7.09E-10 | 1.83E-09 | none | intergenic_variant | 16:73298565 |  |
| BGP | 16 | 62130142 | C | A | 0.015 | 0.86 | 0.17 | 4.17E-07 | 4.95E-07 | 7.82E-07 | none | intergenic_variant | 16:62130142 |  |
| LDL | 14 | 72392076 | A | C | 0.016 | -0.88 | 0.17 | 3.23E-07 | 3.60E-07 | 5.47E-07 | none | intergenic_variant | 14:72392076 |  |
| RG | 11 | 62752440 | A | G | 0.014 | -0.94 | 0.18 | 2.58E-07 | 2.59E-07 | 3.67E-07 | none | 5_prime_UTR_variant | 11:62752440 | SLC22A6 |
| RG | 17 | 10918336 | A | C | 0.027 | 0.71 | 0.13 | 8.25E-08 | 1.16E-07 | 2.27E-07 | none | intergenic_variant | 17:10918336 |  |
| HDL | 3 | 4234628 | A | C | 0.023 | 0.79 | 0.14 | 2.44E-08 | 2.43E-08 | 3.79E-08 | none | intron_variant | 3:4234628 | SUMF1 |
| HGB | 15 | 56731644 | T | TA | 0.043 | 0.67 | 0.12 | 1.41E-08 | 1.35E-08 | 2.41E-08 | none | intron_variant | rs35812910 | TEX9;MNS1 |
| HCT | 15 | 56731644 | T | TA | 0.043 | 0.69 | 0.12 | 6.24E-09 | 6.00E-09 | 1.14E-08 | none | intron_variant | rs35812910 | TEX9;MNS1 |
| HDL | 11 | 116701353 | T | C | 0.019 | 1.03 | 0.16 | 1.52E-10 | 1.65E-10 | 3.72E-10 | HYPERALPHALIPOPROTEINEMIA 2 | stop_gained;splice_region_variant | rs76353203 | APOC3 |
| HDL | 16 | 66758967 | T | C | 0.018 | -1.1 | 0.16 | 1.09E-11 | 1.41E-11 | 4.31E-11 | none | intron_variant | chr16:66758967 | DYNC1LI2;RP11-63M22.2 |
| TG | 11 | 116701353 | T | C | 0.019 | -1.1 | 0.16 | 1.08E-11 | 1.16E-11 | 3.16E-11 | HYPERALPHALIPOPROTEINEMIA 2 | stop_gained;splice_region_variant | rs76353203 | APOC3 |
| TSH | 2 | 228802502 | G | A | 0.336 | -0.24 | 0.05 | 5.01E-07 | 4.91E-07 | 6.73E-07 | none | intergenic_variant | 2:228802502 |  |
| WHR | 11 | 44108888 | C | T | 0.358 | -0.24 | 0.05 | 4.17E-07 | 4.74E-07 | 7.62E-07 | none | downstream_gene_variant | 11:44108888 |  |
| WaistBMIadj | 5 | 143052223 | A | G | 0.208 | -0.29 | 0.06 | 3.40E-07 | 4.24E-07 | 7.39E-07 | none | non_coding_transcript_exon_variant | 5:143052223 | CTB-57H20.1 |
| FI | 11 | 18054777 | G | A | 0.367 | -0.28 | 0.05 | 3.66E-07 | 4.04E-07 | 7.11E-07 | none | intron_variant | 11:18054777 | TPH1 |
| adiponectinBMIadj | 3 | 6390337 | C | T | 0.197 | 0.32 | 0.06 | 3.74E-07 | 3.70E-07 | 5.64E-07 | none | intron_variant | rs1435723 | AC026167.1 |
| HGB | 3 | 168885028 | C | T | 0.126 | -0.37 | 0.07 | 3.25E-07 | 3.39E-07 | 5.27E-07 | none | intron_variant | rs9809961 | MECOM |
| LPCR | 16 | 77938928 | G | C | 0.281 | 0.28 | 0.05 | 3.52E-07 | 3.38E-07 | 4.98E-07 | none | intron_variant | 16:77938928 | VAT1L |
| WaistBMIadj | 2 | 209253879 | G | A | 0.142 | 0.35 | 0.07 | 3.20E-07 | 3.09E-07 | 4.53E-07 | none | intron_variant | 2:209253879 | PTH2R |
| MCHC | 11 | 47377526 | A | AC | 0.314 | -0.27 | 0.05 | 2.72E-07 | 2.62E-07 | 3.90E-07 | none | double allele | rs142497874 | SPI1 |
| LDL | 10 | 36066439 | T | A | 0.438 | 0.23 | 0.04 | 2.20E-07 | 2.50E-07 | 3.86E-07 | none | intergenic_variant | rs10764055 |  |
| MID | 16 | 81582440 | C | G | 0.22 | -0.29 | 0.06 | 2.33E-07 | 2.24E-07 | 3.36E-07 | none | intron_variant | 16:81582440 | CMIP |
| TC | 20 | 16482169 | T | C | 0.118 | 0.34 | 0.06 | 1.77E-07 | 1.89E-07 | 2.84E-07 | none | intron_variant | 20:16482169 | KIF16B |
| HipBMIadj | 1 | 170198941 | A | G | 0.074 | -0.48 | 0.09 | 1.68E-07 | 1.89E-07 | 3.22E-07 | none | intron_variant | 1:170198941 | RP11-297H3.3 |
| Height | 8 | 116052374 | G | C | 0.096 | 0.42 | 0.08 | 1.48E-07 | 1.50E-07 | 2.34E-07 | none | intergenic_variant | 8:116052374 |  |
| LYM | 17 | 77951023 | T | C | 0.257 | 0.29 | 0.06 | 1.41E-07 | 1.47E-07 | 2.37E-07 | none | intron_variant | 17:77951023 | TBC1D16 |
| TSH | 10 | 91092558 | A | T | 0.239 | 0.27 | 0.05 | 1.46E-07 | 1.42E-07 | 2.02E-07 | none | intron_variant | 10:91092558 | LIPA;IFIT3 |
| TSH | 3 | 193922574 | T | G | 0.146 | -0.34 | 0.06 | 1.13E-07 | 1.20E-07 | 1.85E-07 | none | intron_variant | 3:193922574 | RP11-513G11.4 |
| LDL | 19 | 45412079 | T | C | 0.079 | -0.42 | 0.08 | 1.08E-07 | 1.07E-07 | 1.56E-07 | Annotated by HGMD but no phenotype description is publicly available;Response to statins (LDL cholesterol change) | missense_variant;coding_sequence_variant | rs7412 | APOE |
| RDW | 12 | 127985024 | C | A | 0.051 | -0.58 | 0.11 | 1.00E-07 | 1.03E-07 | 1.84E-07 | none | intergenic_variant | 12:127985024 |  |
| SGP | 9 | 27869510 | A | G | 0.089 | -0.4 | 0.07 | 8.65E-08 | 1.01E-07 | 1.68E-07 | none | intergenic_variant | rs2383733 |  |
| adiponectin | 4 | 73880050 | C | G | 0.202 | -0.3 | 0.06 | 8.48E-08 | 8.93E-08 | 1.43E-07 | none | intergenic_variant | rs62309628 |  |
| WBC | 9 | 3624557 | A | G | 0.212 | 0.32 | 0.06 | 5.52E-08 | 8.04E-08 | 1.81E-07 | none | intron_variant | 9:3624557 | RP11-509J21.1 |
| SBPBMIadj | 10 | 109676659 | A | AG | 0.486 | -0.32 | 0.06 | 6.89E-08 | 6.75E-08 | 1.52E-07 | none | intron_variant | rs112349650 | RP11-215N21.1 |
| TC | 5 | 37915720 | A | G | 0.314 | -0.25 | 0.05 | 6.87E-08 | 6.66E-08 | 9.72E-08 | none | intron_variant | rs4574573 | CTD-2194L12.2 |
| HCT | 3 | 168885028 | C | T | 0.126 | -0.4 | 0.07 | 4.47E-08 | 4.75E-08 | 8.47E-08 | none | intron_variant | rs9809961 | MECOM |
| FI | 4 | 72869003 | C | A | 0.339 | -0.32 | 0.06 | 4.74E-08 | 4.52E-08 | 8.43E-08 | none | intergenic_variant | 4:72869003 |  |
| RDW | 9 | 24017665 | G | A | 0.338 | -0.29 | 0.05 | 4.38E-08 | 4.42E-08 | 7.45E-08 | none | intergenic_variant | rs35456165 |  |
| RBC | 10 | 131993 | A | G | 0.135 | -0.39 | 0.07 | 3.30E-08 | 3.73E-08 | 6.83E-08 | none | intron_variant | 10:131993 | IL9RP2 |
| SBP | 10 | 109676659 | A | AG | 0.481 | -0.33 | 0.06 | 2.30E-08 | 2.32E-08 | 5.76E-08 | none | intron_variant | rs112349650 | RP11-215N21.1 |
| RDW | 17 | 54621347 | C | T | 0.096 | -0.46 | 0.08 | 1.59E-08 | 1.54E-08 | 2.90E-08 | none | intergenic_variant | 17:54621347 |  |
| LDL | 20 | 16482169 | T | C | 0.118 | 0.38 | 0.06 | 3.05E-09 | 3.41E-09 | 6.60E-09 | none | intron_variant | 20:16482169 | KIF16B |
| TG | 1 | 237565708 | A | AT | 0.179 | 0.33 | 0.06 | 3.05E-09 | 2.93E-09 | 5.05E-09 | none | intron_variant | rs200918659 | RYR2 |
| HDL | 16 | 57004889 | A | G | 0.417 | 0.33 | 0.04 | 8.72E-15 | 9.40E-15 | 4.74E-14 | Triglycerides | intron_variant | rs7205804 | CETP |
| Bilirubin | 2 | 234664586 | A | ATC | 0.287 | 0.44 | 0.05 | 3.84E-20 | 5.21E-20 | 1.41E-18 | none | non coding transcript exon variant | rs35754645 | UGT1A8;UGT1A10;UGT1A9;UGT1A7;UGT1A6;UGT1A5;UGT1A4;UGT1A3 |

**b. 1x WGS**

| **trait** | **chr** | **ps** | **minor** | **major** | **MAF** | **beta** | **se** | **Wald test p-value** | **LR p-value** | **score test p-value** | **previous associations** | **ensembl most severe consequence** | **rsID if applicable** | **mapped gene if applicable** |
| --- | --- | --- | --- | --- | --- | --- | --- | --- | --- | --- | --- | --- | --- | --- |
| WHR | 22 | 20747018 | T | C | 0.003 | 2.09 | 0.41 | 3.15E-07 | 3.24E-07 | 4.90E-07 | none | upstream_gene_variant | rs1025878347 |  |
| HGB | 11 | 8010293 | G | A | 0.007 | -1.49 | 0.29 | 2.45E-07 | 2.44E-07 | 3.68E-07 | none | intron_variant | rs11041700 | EIF3F |
| adiponectin | 18 | 55257281 | T | C | 0.006 | -1.41 | 0.27 | 2.27E-07 | 2.27E-07 | 3.42E-07 | none | upstream_gene_variant | rs75861770 |  |
| MCHC | 4 | 161522850 | T | C | 0.006 | -1.54 | 0.29 | 2.12E-07 | 2.04E-07 | 3.11E-07 | none | intergenic_variant | rs75097797 |  |
| leptin | 3 | 196163276 | C | A | 0.007 | 1.47 | 0.28 | 1.91E-07 | 1.84E-07 | 2.80E-07 | none | upstream_gene_variant | novel |  |
| Height | 6 | 43216952 | G | A | 0.006 | 1.48 | 0.28 | 1.45E-07 | 1.41E-07 | 2.18E-07 | none | intron_variant | novel | TTBK1 |
| RG | 8 | 18633903 | G | T | 0.003 | -2.15 | 0.4 | 1.22E-07 | 1.24E-07 | 1.86E-07 | none | intron_variant | rs978165175;rs142207507 | PSD3 |
| HDL | 2 | 55373609 | A | C | 0.006 | -1.42 | 0.27 | 1.22E-07 | 1.20E-07 | 1.73E-07 | none | intergenic_variant | rs540995230 |  |
| Ferritin | 6 | 71377781 | A | C | 0.006 | -1.47 | 0.28 | 1.02E-07 | 1.02E-07 | 1.54E-07 | none | missense_variant | rs112439957 | SMAP1 |
| RG | 19 | 57109371 | T | C | 0.002 | -2.41 | 0.44 | 6.15E-08 | 6.39E-08 | 1.00E-07 | none | intron_variant | rs535491193 | ZNF71 |
| Height | 1 | 95192620 | A | G | 0.009 | -1.27 | 0.23 | 4.87E-08 | 4.86E-08 | 7.94E-08 | none | intron_variant | rs145267153 | LINC01057 |
| gammaGT | 4 | 3875226 | A | G | 0.009 | 1.23 | 0.22 | 4.40E-08 | 4.25E-08 | 6.42E-08 | none | intergenic_variant | rs74608440 |  |
| adiponectinBMIadj | 18 | 55257281 | T | C | 0.007 | -1.63 | 0.28 | 1.03E-08 | 1.12E-08 | 2.50E-08 | none | upstream_gene_variant | rs75861770 |  |
| HDL | 16 | 64898622 | G | T | 0.004 | -1.9 | 0.31 | 1.74E-09 | 1.67E-09 | 2.97E-09 | none | intergenic_variant | novel |  |
| TG | 11 | 116701354 | A | G | 0.01 | -1.33 | 0.2 | 7.43E-11 | 7.88E-11 | 1.74E-10 | HYPERALPHALIPOPROTEINEMIA 2 | splice_donor_variant | rs138326449 | APOC3 |
| HDL | 16 | 69035884 | C | A | 0.008 | -1.74 | 0.23 | 1.56E-13 | 1.48E-13 | 5.26E-13 | none | intron_variant | rs370683912 | TANGO6 |
| HDL | 16 | 70756259 | T | C | 0.007 | -1.81 | 0.24 | 6.32E-14 | 5.99E-14 | 2.32E-13 | none | intron_variant | rs188252530 | VAC14 |
| HDL | 16 | 73298565 | C | T | 0.01 | -1.6 | 0.21 | 1.46E-14 | 1.37E-14 | 6.06E-14 | none | intergenic_variant | novel |  |
| HDL | 16 | 66378081 | T | C | 0.009 | -1.79 | 0.22 | 1.10E-15 | 1.03E-15 | 5.86E-15 | none | regulatory_region_variant | rs151205117 |  |
| Bilirubin | 11 | 4437986 | A | G | 0.014 | 0.92 | 0.18 | 3.23E-07 | 3.16E-07 | 4.38E-07 | none | upstream_gene_variant | rs148016671 |  |
| adiponectin | 4 | 29843613 | C | T | 0.038 | -0.57 | 0.11 | 3.13E-07 | 3.05E-07 | 4.32E-07 | none | intergenic_variant | rs34891642 |  |
| TC | 19 | 11388909 | T | G | 0.028 | -0.63 | 0.12 | 2.79E-07 | 2.78E-07 | 3.92E-07 | none | intergenic_variant | rs151018814 |  |
| PDW | 2 | 197713430 | C | T | 0.037 | 0.61 | 0.12 | 2.89E-07 | 2.78E-07 | 4.11E-07 | none | intron_variant | rs79078472 | PGAP1 |
| HDL | 3 | 4234628 | A | C | 0.023 | 0.71 | 0.14 | 2.69E-07 | 2.71E-07 | 3.81E-07 | none | intron_variant | rs138829006 | SUMF1 |
| LPCR | 2 | 197713430 | C | T | 0.037 | 0.61 | 0.12 | 2.50E-07 | 2.41E-07 | 3.61E-07 | none | intron_variant | rs79078472 | PGAP1 |
| leptin | 19 | 32030022 | A | T | 0.015 | -0.99 | 0.19 | 2.44E-07 | 2.39E-07 | 3.65E-07 | none | upstream_gene_variant | rs142827020 |  |
| RDW | 16 | 571731 | A | G | 0.011 | -1.13 | 0.22 | 2.43E-07 | 2.36E-07 | 3.55E-07 | none | 3_prime_UTR_variant | rs183291067 | RAB11FIP3 |
| MID | 3 | 108401373 | G | A | 0.05 | -0.55 | 0.11 | 2.16E-07 | 2.08E-07 | 3.17E-07 | none | intron_variant | rs116392621 | DZIP3 |
| BGP | 18 | 26248392 | T | A | 0.046 | -0.51 | 0.1 | 2.01E-07 | 1.95E-07 | 2.73E-07 | none | intergenic_variant | rs112013002;rs1628183;rs771139413 |  |
| TC | 14 | 72392076 | A | C | 0.016 | -0.88 | 0.17 | 1.89E-07 | 1.84E-07 | 2.56E-07 | none | intergenic_variant | novel |  |
| Fe_iron | 13 | 53763262 | A | G | 0.015 | 0.91 | 0.17 | 1.52E-07 | 1.48E-07 | 2.12E-07 | none | intergenic_variant | rs116253332 |  |
| LYM | 11 | 8059760 | A | G | 0.025 | -0.79 | 0.15 | 1.14E-07 | 1.17E-07 | 1.91E-07 | none | intron_variant | rs36206809 | TUB;RP11-236J17.6 |
| BGP | 12 | 69338638 | C | G | 0.05 | 0.52 | 0.1 | 8.18E-08 | 8.59E-08 | 1.38E-07 | none | intron_variant | rs117743406 | CPM |
| TSH | 19 | 22796761 | T | C | 0.035 | -0.59 | 0.11 | 8.84E-08 | 8.56E-08 | 1.25E-07 | none | downstream_gene_variant | rs35954710 |  |
| LDL | 14 | 72392076 | A | C | 0.016 | -0.9 | 0.17 | 7.84E-08 | 7.58E-08 | 1.10E-07 | none | intergenic_variant | novel |  |
| adiponectin | 1 | 235746578 | A | G | 0.036 | -0.6 | 0.11 | 7.33E-08 | 7.09E-08 | 1.09E-07 | none | intron_variant | rs115072523 | GNG4 |
| RG | 2 | 85968805 | A | C | 0.044 | -0.55 | 0.1 | 6.94E-08 | 6.73E-08 | 9.93E-08 | none | regulatory_region_variant | rs116010068 |  |
| TG | 17 | 55839278 | T | C | 0.031 | 0.64 | 0.12 | 6.93E-08 | 6.71E-08 | 9.79E-08 | none | regulatory_region_variant | rs9912215 |  |
| BGP | 16 | 62130142 | C | A | 0.016 | 0.85 | 0.16 | 6.91E-08 | 6.68E-08 | 9.84E-08 | none | intergenic_variant | rs28408198 |  |
| Height | 15 | 26279796 | A | G | 0.014 | -1.05 | 0.19 | 3.74E-08 | 3.71E-08 | 6.14E-08 | none | intron_variant | rs189010705 | RP11-1084I9.1 |
| HGB | 15 | 56719785 | C | T | 0.043 | 0.62 | 0.11 | 2.86E-08 | 2.73E-08 | 4.65E-08 | none | intron_variant | rs553450981;rs795784;rs774475285 | TEX9;MNS1 |
| BGP | 7 | 135326332 | C | T | 0.041 | -0.59 | 0.1 | 2.75E-08 | 2.69E-08 | 4.18E-08 | none | intron_variant | rs117784919 | NUP205 |
| MCV | 16 | 571731 | A | G | 0.011 | -1.28 | 0.23 | 2.37E-08 | 2.29E-08 | 4.01E-08 | none | 3_prime_UTR_variant | rs183291067 | RAB11FIP3 |
| FT4 | 5 | 19313119 | C | A | 0.021 | 0.78 | 0.14 | 1.75E-08 | 1.72E-08 | 2.77E-08 | none | intergenic_variant | novel |  |
| HCT | 15 | 56719785 | C | T | 0.044 | 0.63 | 0.11 | 1.25E-08 | 1.19E-08 | 2.15E-08 | none | intron_variant | rs553450981;rs795784;rs774475285 | TEX9;MNS1 |
| MCH | 16 | 571731 | A | G | 0.011 | -1.37 | 0.23 | 2.73E-09 | 2.60E-09 | 5.26E-09 | none | 3_prime_UTR_variant | rs183291067 | RAB11FIP3 |
| RG | 11 | 62752440 | A | G | 0.013 | -1.15 | 0.18 | 6.96E-10 | 6.73E-10 | 1.30E-09 | none | 5_prime_UTR_variant | rs57677322 | SLC22A6 |
| RG | 17 | 10902383 | G | C | 0.027 | 0.81 | 0.13 | 1.97E-10 | 1.94E-10 | 4.15E-10 | none | intergenic_variant | rs76050199 |  |
| HDL | 11 | 116701353 | T | C | 0.014 | 1.24 | 0.17 | 3.30E-13 | 3.21E-13 | 1.10E-12 | HYPERALPHALIPOPROTEINEMIA 2 | stop_gained;splice_region_variant | rs76353203;CM086325 | APOC3 |
| TG | 1 | 237565709 | A | T | 0.205 | 0.26 | 0.05 | 3.44E-07 | 3.34E-07 | 4.53E-07 | none | intron_variant | rs753027291;rs12041884;rs200918659 | RYR2 |
| TG | 2 | 134346210 | C | T | 0.289 | -0.23 | 0.04 | 3.37E-07 | 3.27E-07 | 4.44E-07 | none | intergenic_variant | rs6430433 |  |
| BMI | 20 | 10411454 | A | T | 0.412 | 0.24 | 0.05 | 2.97E-07 | 3.00E-07 | 4.70E-07 | none | intron_variant | rs6077785 | MKKS |
| SGP | 1 | 18197967 | C | G | 0.472 | 0.23 | 0.04 | 2.80E-07 | 2.72E-07 | 3.78E-07 | none | intergenic_variant | rs618124 |  |
| WaistBMIadj | 15 | 66483776 | C | T | 0.085 | 0.43 | 0.08 | 2.73E-07 | 2.71E-07 | 4.07E-07 | none | intron_variant | rs28702433 | MEGF11 |
| HGB | 5 | 34347349 | G | A | 0.376 | 0.24 | 0.05 | 2.81E-07 | 2.70E-07 | 4.01E-07 | none | intergenic_variant | rs7726646 |  |
| BMI | 14 | 81134882 | T | C | 0.099 | -0.4 | 0.08 | 2.28E-07 | 2.69E-07 | 4.92E-07 | none | intron_variant | rs114068807 | CEP128 |
| gammaGT | 10 | 55148854 | G | A | 0.43 | -0.22 | 0.04 | 2.44E-07 | 2.37E-07 | 3.29E-07 | none | intergenic_variant | rs7082340 |  |
| FT4 | 13 | 103953533 | A | G | 0.059 | 0.47 | 0.09 | 2.43E-07 | 2.37E-07 | 3.30E-07 | none | intergenic_variant | rs146017506 |  |
| TSH | 3 | 193916181 | A | G | 0.205 | -0.27 | 0.05 | 2.28E-07 | 2.21E-07 | 3.08E-07 | none | downstream_gene_variant | rs59381142 |  |
| WaistBMIadj | 11 | 44108888 | C | T | 0.343 | -0.25 | 0.05 | 2.14E-07 | 2.09E-07 | 3.15E-07 | none | downstream_gene_variant | rs7115682 |  |
| MPV | 12 | 125082675 | A | C | 0.052 | 0.56 | 0.11 | 1.81E-07 | 1.81E-07 | 2.81E-07 | none | regulatory_region_variant | rs12423958 |  |
| TC | 2 | 101380915 | G | A | 0.285 | -0.24 | 0.05 | 1.80E-07 | 1.81E-07 | 2.58E-07 | none | intergenic_variant | rs6746018 |  |
| FI | 11 | 18044648 | G | A | 0.356 | -0.28 | 0.05 | 1.72E-07 | 1.64E-07 | 2.77E-07 | none | non_coding_transcript_exon_variant | rs10741734 | TPH1 |
| PDW | 12 | 125082675 | A | C | 0.052 | 0.56 | 0.11 | 1.60E-07 | 1.59E-07 | 2.47E-07 | none | regulatory_region_variant | rs12423958 |  |
| MPV | 19 | 8970903 | T | C | 0.101 | -0.4 | 0.08 | 1.42E-07 | 1.59E-07 | 2.73E-07 | none | intron_variant | rs4804087 | MUC16 |
| MPV | 2 | 228619651 | T | C | 0.059 | 0.52 | 0.1 | 1.52E-07 | 1.52E-07 | 2.40E-07 | none | intergenic_variant | rs62189960 |  |
| adiponectinBMIadj | 7 | 25451594 | A | T | 0.393 | -0.26 | 0.05 | 1.53E-07 | 1.52E-07 | 2.54E-07 | none | intergenic_variant | rs6953343 |  |
| LPCR | 2 | 228619651 | T | C | 0.059 | 0.52 | 0.1 | 1.47E-07 | 1.48E-07 | 2.34E-07 | none | intergenic_variant | rs62189960 |  |
| Ferritin | 15 | 33668251 | G | T | 0.067 | 0.43 | 0.08 | 1.51E-07 | 1.46E-07 | 2.08E-07 | none | intron_variant | rs568235767 | RYR3 |
| TC | 1 | 223480141 | C | G | 0.171 | 0.28 | 0.05 | 1.43E-07 | 1.40E-07 | 1.98E-07 | none | intron_variant | rs1772271 | SUSD4 |
| HGB | 3 | 168948198 | C | T | 0.11 | -0.4 | 0.07 | 1.42E-07 | 1.37E-07 | 2.11E-07 | none | intron_variant | rs75771628 | MECOM |
| Fe_iron | 7 | 105676297 | G | A | 0.073 | -0.43 | 0.08 | 1.39E-07 | 1.34E-07 | 1.92E-07 | none | 3_prime_UTR_variant | rs144514220 | CDHR3 |
| PCT | 20 | 840950 | G | C | 0.067 | -0.48 | 0.09 | 1.25E-07 | 1.26E-07 | 2.00E-07 | none | downstream_gene_variant | rs74531087 |  |
| PDW | 3 | 86947782 | A | T | 0.299 | -0.27 | 0.05 | 1.17E-07 | 1.14E-07 | 1.81E-07 | none | intergenic_variant | rs9880801 |  |
| RDW | 17 | 54621347 | C | T | 0.093 | -0.42 | 0.08 | 1.03E-07 | 1.01E-07 | 1.59E-07 | none | intergenic_variant | rs72835517 |  |
| HipBMIadj | 1 | 170198941 | A | G | 0.072 | -0.48 | 0.09 | 8.91E-08 | 9.63E-08 | 1.64E-07 | none | intron_variant | rs7521954 | RP11-297H3.3 |
| TSH | 10 | 91092706 | C | T | 0.242 | 0.26 | 0.05 | 9.91E-08 | 9.59E-08 | 1.39E-07 | none | intron_variant | rs4630205 | LIPA;IFIT3 |
| MCV | 10 | 36305596 | C | T | 0.141 | -0.35 | 0.07 | 9.04E-08 | 8.69E-08 | 1.40E-07 | none | intergenic_variant | rs3851069 |  |
| adiponectin | 12 | 45861909 | C | G | 0.495 | 0.23 | 0.04 | 8.79E-08 | 8.46E-08 | 1.28E-07 | none | intergenic_variant | rs4603367 |  |
| leptin | 8 | 68464358 | C | G | 0.285 | 0.27 | 0.05 | 8.66E-08 | 8.33E-08 | 1.32E-07 | none | intron_variant | rs924741 | CPA6 |
| FT4 | 7 | 152700215 | G | A | 0.187 | 0.29 | 0.05 | 8.11E-08 | 7.86E-08 | 1.15E-07 | none | intergenic_variant | rs11486922 |  |
| LPCR | 12 | 125082675 | A | C | 0.052 | 0.57 | 0.11 | 7.84E-08 | 7.72E-08 | 1.25E-07 | none | regulatory_region_variant | rs12423958 |  |
| leptin | 14 | 58047905 | T | C | 0.062 | 0.5 | 0.09 | 7.32E-08 | 7.31E-08 | 1.19E-07 | none | non_coding_transcript_exon_variant | rs117786916 | SLC35F4;RP11-409I10.2 |
| MCHC | 11 | 47393638 | T | C | 0.311 | -0.27 | 0.05 | 7.57E-08 | 7.25E-08 | 1.17E-07 | Fasting proinsulin | intron_variant | rs4992357 | SPI1 |
| FI | 4 | 72869003 | C | A | 0.337 | -0.31 | 0.06 | 7.33E-08 | 6.97E-08 | 1.25E-07 | none | intergenic_variant | rs4263362 |  |
| TC | 5 | 37915720 | A | G | 0.313 | -0.24 | 0.04 | 6.56E-08 | 6.53E-08 | 9.70E-08 | none | intron_variant | rs4574573 | CTD-2194L12.2 |
| Weight | 3 | 193909907 | T | C | 0.24 | 0.29 | 0.05 | 5.34E-08 | 5.12E-08 | 8.15E-08 | none | intergenic_variant | rs143707274 |  |
| LDL | 19 | 45412079 | T | C | 0.086 | -0.4 | 0.07 | 5.14E-08 | 4.98E-08 | 7.41E-08 | Response to statins (LDL cholesterol change) | missense_variant;coding_sequence_variant | rs7412;CM860003 | APOE |
| PLT | 13 | 65366000 | T | G | 0.395 | 0.26 | 0.05 | 4.57E-08 | 4.49E-08 | 7.53E-08 | Coronary Artery Disease | intergenic_variant | rs9540221 |  |
| RBC | 16 | 434104 | T | G | 0.065 | 0.51 | 0.09 | 3.66E-08 | 4.23E-08 | 7.90E-08 | none | intron_variant | rs72765895 | TMEM8A;Z97634.3 |
| HCT | 3 | 168885028 | C | T | 0.127 | -0.39 | 0.07 | 3.35E-08 | 3.20E-08 | 5.41E-08 | none | intron_variant | rs9809961 | MECOM |
| adiponectinBMIadj | 3 | 6390337 | C | T | 0.195 | 0.34 | 0.06 | 2.79E-08 | 2.68E-08 | 4.78E-08 | none | intron_variant | rs1435723 | AC026167.1 |
| TC | 20 | 16492242 | T | C | 0.118 | 0.36 | 0.06 | 2.22E-08 | 2.15E-08 | 3.34E-08 | none | intron_variant | rs6135772 | KIF16B |
| HipBMIadj | 6 | 33791230 | T | C | 0.346 | 0.27 | 0.05 | 1.87E-08 | 1.88E-08 | 3.54E-08 | none | intergenic_variant | rs10947443 |  |
| Height | 8 | 116052374 | G | C | 0.092 | 0.43 | 0.07 | 1.38E-08 | 1.40E-08 | 2.52E-08 | none | intergenic_variant | rs71528491 |  |
| leptin | 18 | 3312574 | T | G | 0.093 | 0.44 | 0.08 | 9.53E-09 | 9.12E-09 | 1.67E-08 | none | intron_variant | rs7238627 | RP11-838N2.5 |
| SGP | 9 | 27869510 | A | G | 0.085 | -0.42 | 0.07 | 6.73E-09 | 6.80E-09 | 1.17E-08 | none | intergenic_variant | rs2383733 |  |
| MPV | 3 | 86947782 | A | T | 0.299 | -0.29 | 0.05 | 6.09E-09 | 5.81E-09 | 1.10E-08 | none | intergenic_variant | rs9880801 |  |
| MIDPC | 14 | 103817631 | C | T | 0.34 | 0.29 | 0.05 | 4.80E-09 | 4.61E-09 | 8.88E-09 | none | intergenic_variant | rs2403168 |  |
| SBPBMIadj | 10 | 109678540 | A | G | 0.506 | 0.34 | 0.06 | 4.72E-09 | 4.50E-09 | 1.35E-08 | none | intron_variant | rs982601001;rs10884572 | RP11-215N21.1 |
| LDL | 10 | 36066439 | T | A | 0.441 | 0.25 | 0.04 | 2.99E-09 | 2.91E-09 | 5.12E-09 | none | intergenic_variant | rs10764055 |  |
| WBC | 9 | 3624557 | A | G | 0.212 | 0.34 | 0.06 | 1.35E-09 | 1.28E-09 | 2.77E-09 | none | intron_variant | rs58276821 | RP11-509J21.1 |
| SBP | 10 | 109678540 | A | G | 0.51 | 0.35 | 0.06 | 1.18E-09 | 1.13E-09 | 3.85E-09 | none | intron_variant | rs982601001;rs10884572 | RP11-215N21.1 |
| LDL | 20 | 16482169 | T | C | 0.119 | 0.39 | 0.06 | 7.80E-10 | 7.51E-10 | 1.41E-09 | none | intron_variant | rs76841054 | KIF16B |
| WaistBMIadj | 5 | 143075283 | G | A | 0.208 | -0.34 | 0.05 | 4.87E-10 | 4.63E-10 | 1.04E-09 | none | intron_variant | rs11749182 | CTB-57H20.1 |
| RBC | 10 | 124767 | T | C | 0.138 | -0.42 | 0.06 | 9.16E-11 | 9.20E-11 | 2.47E-10 | none | upstream_gene_variant | rs9419461 |  |
| HDL | 16 | 57005479 | A | C | 0.417 | 0.35 | 0.04 | 2.66E-17 | 2.51E-17 | 2.15E-16 | Triglycerides | intron_variant | rs1532624 | CETP |
| Bilirubin | 2 | 234673309 | T | C | 0.287 | 0.45 | 0.04 | 1.53E-22 | 1.62E-22 | 8.45E-21 | Total bilirubin levels in HIV-1 infection | intron_variant | rs4148325 | UGT1A8;UGT1A10;UGT1A9;UGT1A7;UGT1A6;UGT1A5;UGT1A4;UGT1A3;UGT1A1 |

**Supplementary Table 3: Independent signals across MAF categories in the 1x and imputed GWAS, before and after filtering for associations significant at 5x10-5 in the 22x WGS dataset.**

|  |  | **rare** | **low-frequency** | **common** |
| --- | --- | --- | --- | --- |
| **post-filtering** | **1x** | 19 | 30 | 59 |
|  | **imputed GWAS** | 8 | 10 | 14 |
|  | **ratio** | **2.3** | **3** | **4.2** |
|  | **1x** | 56 | 35 | 91 |
| **pre-filtering** | **imputed GWAS** | 130 | 118 | 214 |
|  | **ratio** | **2.3** | **3.4** | **2.3** |

**Supplementary Table 4: Phenotypes used in the association analysis.**

| **Trait name** | **Description** | **Unit** | **Transformation** | **covariates** | **exclusion** |
| --- | --- | --- | --- | --- | --- |
| adiponectinBMIadj | adiponectin adjusted for BMI | ug/mL | inverse normal | age, age^2, BMI | +/- 5 S.D |
| adiponectin | adiponectin | ug/mL | inverse normal | age, age^2 | +/- 5 S.D |
| BGP | osteocalcin | mg/ml | inverse normal | age, age^2 | +/- 5 S.D |
| Bilirubin | bilirubin | mg/dl | inverse normal | age, age^2 | +/- 5 S.D |
| BMI | Body Mass Index | kg/m^2 | inverse normal | age, age^2 | >4 S.D |
| CRP | C-reactive protein | mg/L | inverse normal | age, age^2 | +/- 3 S.D and <0.1, >10 |
| DBPBMIadj | diastolic blood pressure adjusted for BMI | mmHg | inverse normal | age, age^2, BMI | +/- 5 S.D |
| Fe_iron | Iron | mmol/L | inverse normal | age, age^2 | +/- 5 S.D |
| Ferritin | Ferritin | pmol/L | inverse normal | age, age^2 | +/- 5 S.D |
| FG | Fasting glucose | mmol/L | inverse normal | age, age^2 | >7 |
| FIBMIadj | Fasting insulin adjusted for BMI | uIU/ml | inverse normal | age, age^2, BMI | +/- 5 S.D |
| FI | Fasting insulin | uIU/ml | inverse normal | age, age^2 | +/- 5 S.D |
| FT4 | Free Thyroxine | ng/dl | inverse normal | age, age^2 | +/- 5 S.D |
| gammaGT | gamma-glutamyltransferase | IU/L | inverse normal | age, age^2 | +/- 5 S.D |
| GRAN | granulocytes | NA | inverse normal | age, age^2 | none |
| GRANPC | granulocytes, percent | NA | inverse normal | age, age^2 | none |
| HCT | haematocrit | % | inverse normal | age, age^2 | +/- 3 S.D |
| HDL | high-density lipoprotein | mmol/L | inverse normal | none | +/- 5 S.D |
| Height | height | cm | inverse normal | age, age^2 | +/- 5 S.D |
| HGB | haemoglobin | g/dl | inverse normal | age, age^2 | +/- 5 S.D |
| HipBMIadj | hip circumference adjusted for BMI | cm | inverse normal | age, age^2, BMI | >4 S.D |
| Hip | hip circumference | cm | inverse normal | age, age^2 | >4 S.D |
| HOMA_bBMIadj | homeostatic model assessment, b-cell function, adjusted for BMI | - | inverse normal | age, age^2, BMI | none |
| HOMA_b | homeostatic model assessment, b-cell function | - | inverse normal | age, age^2 | none |
| HOMA_irBMIadj | homeostatic model assessment, insulin resistance, adjusted for BMI | - | inverse normal | age, age^2, BMI | none |
| HOMA_ir | homeostatic model assessment, insulin resistance | - | inverse normal | age, age^2 | none |
| LDL | low-density lipoprotein | mmol/L | inverse normal | none | +/- 5 S.D |
| leptin | leptin | ng/mL | inverse normal | age, age^2 | +/- 5 S.D |
| LPCR | large platelet concentration ratio | - | inverse normal | age, age^2 | none |
| LYM | lymphocytes | - | inverse normal | age, age^2 | none |
| LYMPC | lymphocytes, percent | % | inverse normal | age, age^2 | none |
| MCHC | mean corpuscular haemoglobin concentration | g/dL | inverse normal | age, age^2 | +/- 3 S.D |
| MCH | mean content of haemoglobin | pg | inverse normal | age, age^2 | +/- 3 S.D |
| MCV | mean corpuscular volume | fl | inverse normal | age, age^2 | +/- 3 S.D |
| MID | monocytes, eosinophils, basophils | - | inverse normal | age, age^2 | none |
| MIDPC | monocytes, eosinophils, basophils, percent | - | inverse normal | age, age^2 | none |
| MPV | mean platelet volume | - | inverse normal | age, age^2 | none |
| PCT | plateletcrit | - | inverse normal | age, age^2 | none |
| PDW | platelet distribution width | - | inverse normal | age, age^2 | none |
| PLT | platelets | 10^9/L | inverse normal | age, age^2 | +/- 3 S.D |
| RBC | red blood cell count | 10^12/L | inverse normal | none | +/- 3 S.D |
| RDW | red cell distribution width | - | inverse normal | age, age^2 | none |
| RDWPC | red cell distribution width, percent | - | inverse normal | age, age^2 | none |
| RG | random glucose | mmol/L | inverse normal | age, age^2 | none |
| RI | random insulin | uIU/ml | inverse normal | age, age^2 | none |
| SBPBMIadj | systolic blood pressure, adjusted for BMI | mmHg | inverse normal | age, age^2, BMI | +/- 5 S.D |
| SBP | systolic blood pressure | mmHg | inverse normal | age, age^2 | +/- 5 S.D |
| SGP | alanine aminotransferase | IU/L | inverse normal | age, age^2 | +/- 5 S.D |
| TC | total cholesterol | mmol/L | inverse normal | none | +/- 5 S.D |
| TG | triglycerides | mmol/L | log | none | +/- 5 S.D |
| TSH | thyroid stimulating hormone | uIU/ml | inverse normal | age, age^2 | +/- 5 S.D |
| WaistBMIadj | waist circumference adjusted for BMI | cm | inverse normal | age, age^2, BMI | >4 S.D |
| Waist | waist circumference | cm | inverse normal | age, age^2 | >4 S.D |
| WBC | white blood cells | 10^9/L | log | age, age^2 | +/- 3 S.D |
| Weight | weight | kg | inverse normal | age, age^2 | >4 S.D |
| WHRBMIadj | waist-hip ratio adjusted for BMI | - | inverse normal | age, age^2, BMI | >4 S.D |
| WHR | waist-hip ratio | - | inverse normal | age, age^2 | >4 S.D |

**Supplementary Text**

**Read alignment and calling**

Following generation of raw reads on the Illumina HiSeq 2000 and HiSeq 2500 sequencing machines, reads were converted from BCL format to BAM format using the Illumina2BAM (https://github.com/wtsi-npg/illumina2bam) software. Illumina2BAM was again used to de-multiplex lanes that had been sequenced so that the tags were isolated from the body of the read, decoded, and could be used to separate out each lane into lanelets containing individual samples from the multiplex library and the PhiX control. The quality scores were then recalibrated using the purity recalibration algorithm (Abnizova, et al., 2010) using the PhiX data for reference. Read mapping was then carried out using the BWA backtrack algorithm version 0.5.10 using the GRCh37 1000 Genomes phase III reference (also known as hs37d5). PCR and optically duplicated reads were marked using Picard MarkDuplicates (http://broadinstitute.github.io/picard).

In order to ensure the quality of the large quantity of BAMs produced for the project, an automatic quality control system was used to reduce the number of data files that required manual intervention. This system was derived from the one originally designed for the UK10K project (http://www.uk10k.org) and used a series of empirically derived thresholds to assess summary metrics calculated from the input BAMs. These thresholds included: percentage of reads mapped; percentage of duplicate reads marked; various statistics measuring INDEL distribution against read cycle and an insert size overlap percentage. Any lane that fell below the “fail” threshold for any of the metrics were excluded; and any lane that did not fall below these thresholds for any of the metrics was given a status of “pass” and allowed to proceed into the later stages of the pipeline.

Passed lanelets were then merged into BAMs corresponding to the libraries for each sample and duplicates were marked again with Picard MarkDuplicates after which they were then merged into BAMs on a per sample basis. Finally sample level bam improvement was carried out using GATK 1.6(DePristo, et al., 2011; McKenna, et al., 2010) and samtools(Christopoulos, 1997) from git commit 72d6457f7f361c323f62bd2d3170980132ba2113. This consisted of re-alignment of reads around known and discovered INDELs followed by base quality score recalibration both using the GATK, lastly samtools calmd was applied and indexes were created. Known INDELs for realignment were taken from Mill Devine and 1000G Gold set and the 1000G phase low coverage set both part of the Broad’s GATK resource bundle version 2.2. Known variants for BQSR were taken from dbSNP 137 also part of the Broad’s resource bundle. The input BAM files are fed into samtools mpileup to create all-sites BCF files, which are piped into bcftools view to create variant-only VCF files containing genotype calls. We split the genome into chunks of 100,000 base pairs, and separate these chunks into SNV and INDEL files. We run GATK UnifiedGenotyper to calculate site-level annotations.

**Variant Quality Score Recalibration**

Variant quality score recalibration was performed using the QualByDepth, HaplotypeScore, MQRankSum, ReadPosRankSum, FS, InbreedingCoeff and DP annotations. Initial runs were performed on GATK v. 2.7.2, final runs were performed using GATK 3.1.1. With identical input, this change in VQSR version had large consequences in the recalibration output (Supplementary Figure 3). For completeness, we tested recalibration using GATK v.3.6 at the time of publication, which uses a different set of recommended annotations (HaplotypeScore was removed from the model, and MQ was added). This yielded very poor performance for the previously optimal run, with an increasing Ti/Tv by tranche curve instead of a strictly decreasing one. We then restored the set of annotations to HaplotypeScore, BaseQRankSum, MQRankSum, ReadPosRankSum, FS and InbreedingCoeff and obtained a very good performance, with a 99.4% sensitivity at the 2.0 Ti/Tv cutoff point, while conserving the 10% type-I error rate. This is a much better sensitivity/specificity compromise than the cut-off chosen at the analysis stage of this work, and highlights the extreme variability of recalibration results for very-low-depth sequencing data.

**Common-variant 1x signals not detected using GWAS and imputation**

As shown in our comparison, 1x WGS detects at least twice, and up to four times as many true common variant signals than imputed GWAS (Supplementary Table 1). We do not expect this is due to higher genotype quality, as imputation is reliable for common variants. If this were true, high-depth WGS p-values would be close to 1x ones, however we observe instead an almost systematic overestimation of association significance. We posit this is due to lower genotype quality. Indeed, if due to a noisier input, genotypes are more inaccurate in the 1x imputation, we expect more genotyping error and hence more variability around the association p-value we would get with perfect genotypes. By selecting for low p-values in our comparison, we are biasing against variants for which small genotyping errors lead to an underestimation of the effect of genotype on phenotype. As shown in Figure , most of the signals confirmed in the 15x data do have a lower p-value in the 1x study than in both the 22x and imputed GWAS, confirming this hypothesis. This slightly decreased imputation accuracy also explains why we see more successfully detected common variant signals in the 1x and the 15x compared to the imputed GWAS. This does not put in question our findings regarding the power of 1x for detecting association, but it does indicate a slightly lower accuracy, which incidentally remains very high for common variants (Figure 2).

**References**

Abnizova, I.*, et al.* Statistical comparison of methods to estimate the error probability in short-read Illumina sequencing. *J Bioinform Comput Biol* 2010;8(3):579-591.

Christopoulos, K.T.D. Minorities in Greece. Kritiki; 1997.

DePristo, M.A.*, et al.* A framework for variation discovery and genotyping using next-generation DNA sequencing data. *Nat Genet* 2011;43(5):491-498.

McKenna, A.*, et al.* The Genome Analysis Toolkit: a MapReduce framework for analyzing next-generation DNA sequencing data. *Genome Res* 2010;20(9):1297-1303.
