# Supplementary material for: Very low-depth whole-genome sequencing in complex trait association studies
Source: Bioinformatics. 2018 Dec 21;35(15):2555–61. doi: 10.1093/bioinformatics/bty1032 (PMC6662288; doi:10.1093/bioinformatics/bty1032)
Supplement: bty1032_Supplementary_Data [file bty1032_supplementary_data.zip › bty1032-suppl_data/Gilly.Revision.Supplementary.pdf]

Supplementary Data

Supplementary Figure 1: Minor allele concordance for genotype refinement pipelines. Chromosome 11 data, no imputation was performed, only positions called by sequencing are evaluated. Missingness is zero for positions included in the reference panel when a panel is present, and zero across all positions when performing reference-free genotype refinement. Pipelines using Beagle v4 with a reference panel are drawn in blue, pipelines not involving Beagle or not using a reference panel are coloured red. 1KG=1000 Genomes phase I reference panel. Details of the pipelines are presented in Supplementary Table 1.

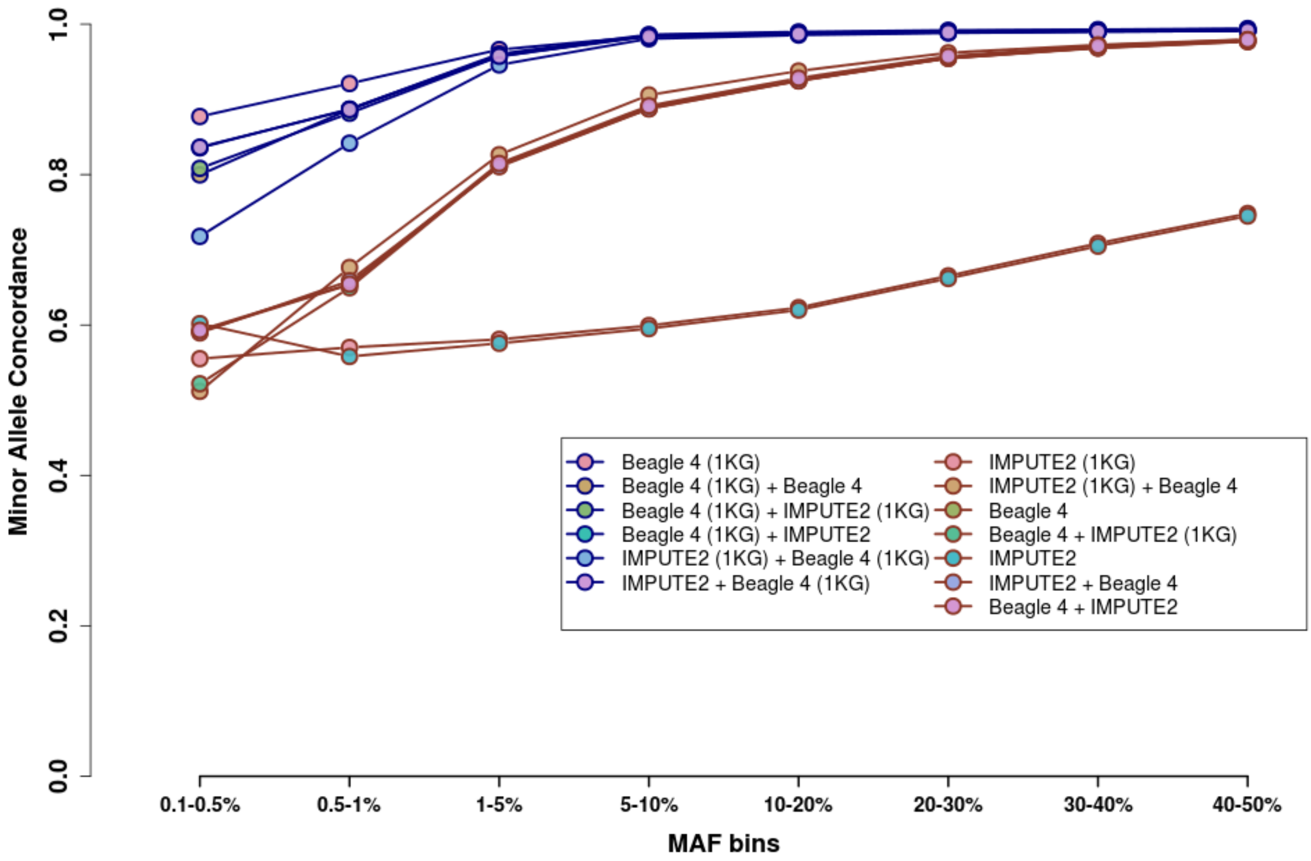

Supplementary Figure 2: Genotype refinement and imputation compute time, measured in core hours, on chunks of 2,000 SNV genome-wide for the MANOLIS cohort. Some chunks located in regions such as the major histocompatibility complex took up to 271 hours (11 days) to complete.

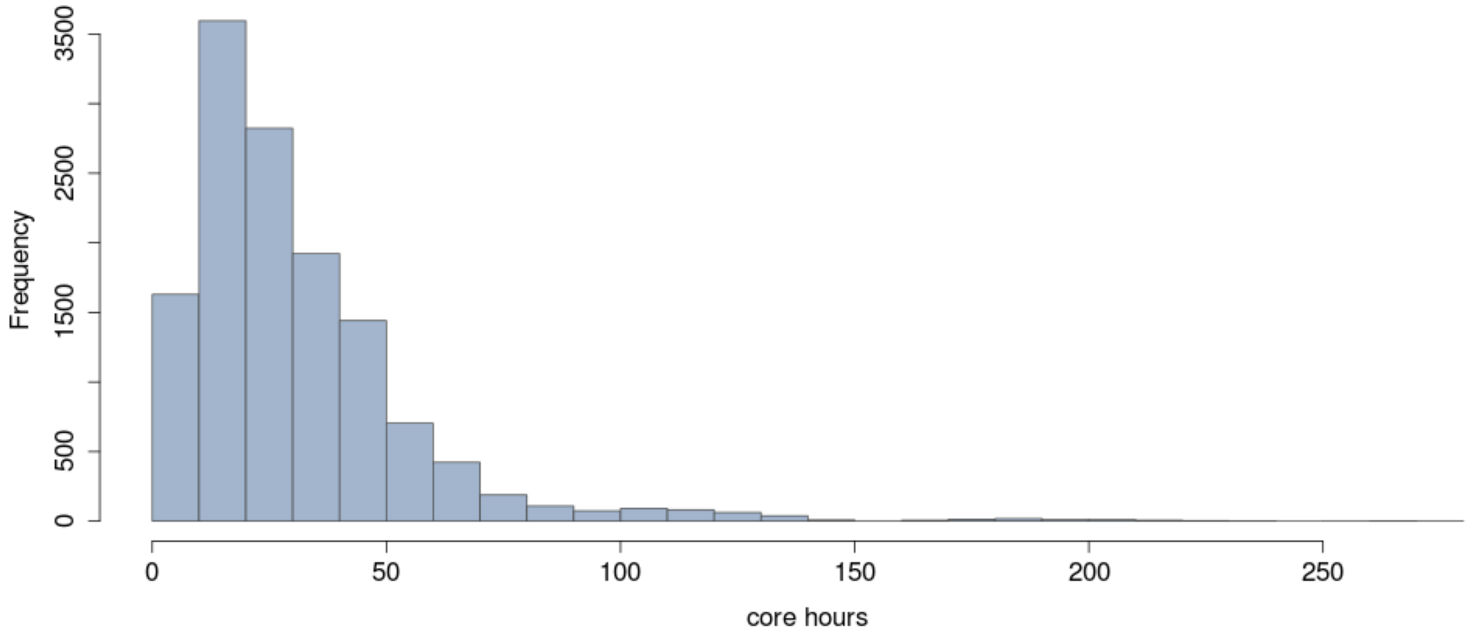

Supplementary Figure 3: Venn diagram of the sequenced and genotyped datasets in MANOLIS. Figure generated using http://bioinformatics.psb.ugent.be/webtools/Venn/

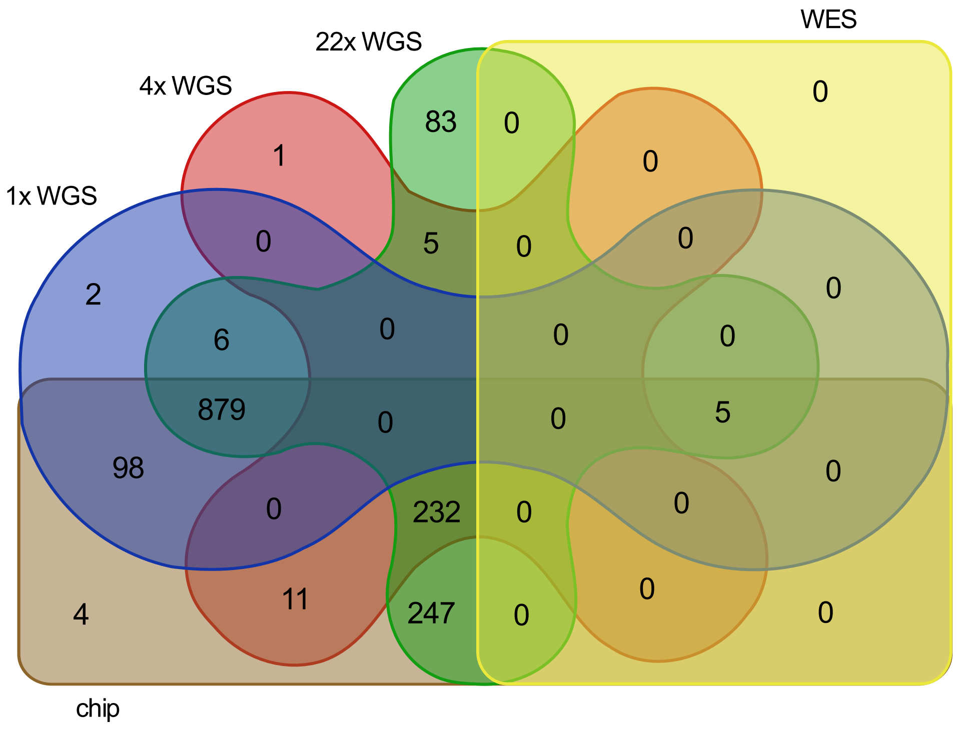

Supplementary Figure 4: VQSR recalibration curves for the MANOLIS cohort. **a.** VQSR version 3.1.1. with default parameters, **b.** using the optimal set of parameters. Ti/Tv ratio for novel variants is given by VQSR. The fraction of true sites called is the proportion of variants in the positive training set called at that tranche or below. The estimated false positive rate is calculated as $FP = \frac{TiTv_{expected} - TiTv_{novel}}{TiTv_{expected} - TiTv_{FP}}$ where $TiTv_{FP} = 0.5$ and $TiTv_{expected}$ is 2.6 for the lower dashed curve, 2.1 for the upper dashed curve, and 2.3 for the solid curve.

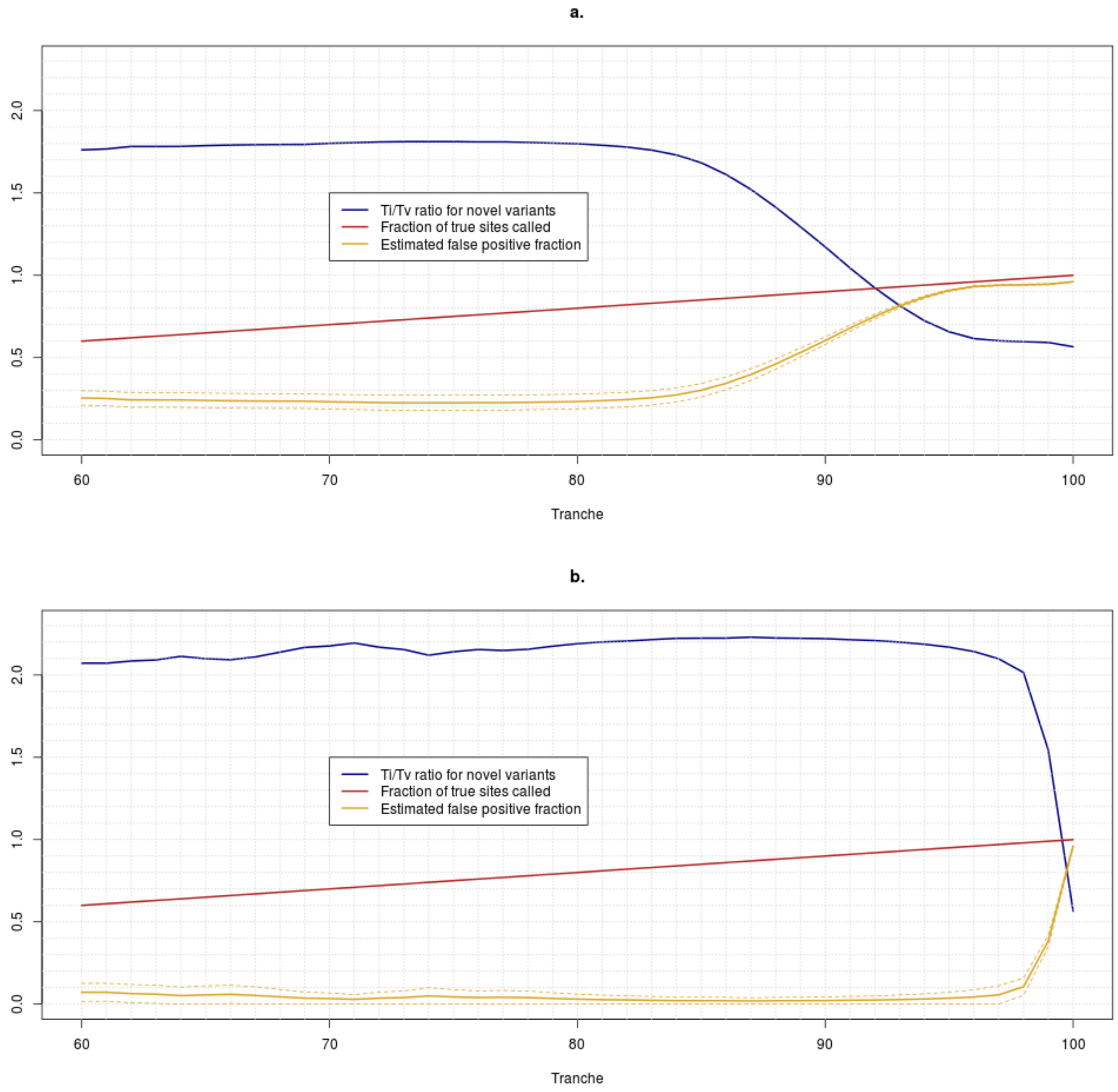

Supplementary Figure 5: VQSR optimisation grid for two versions of VQSR. a. version 2.7.2, b. version 3.1.1. The bottom axis represents the minimum number of variants in the negative training set (minNumBadVariants), the vertical axis is the quality field threshold (qualThreshold) and the top axis is the maximum number of Gaussian distributions in the mixture model (maxGaussians).

a.

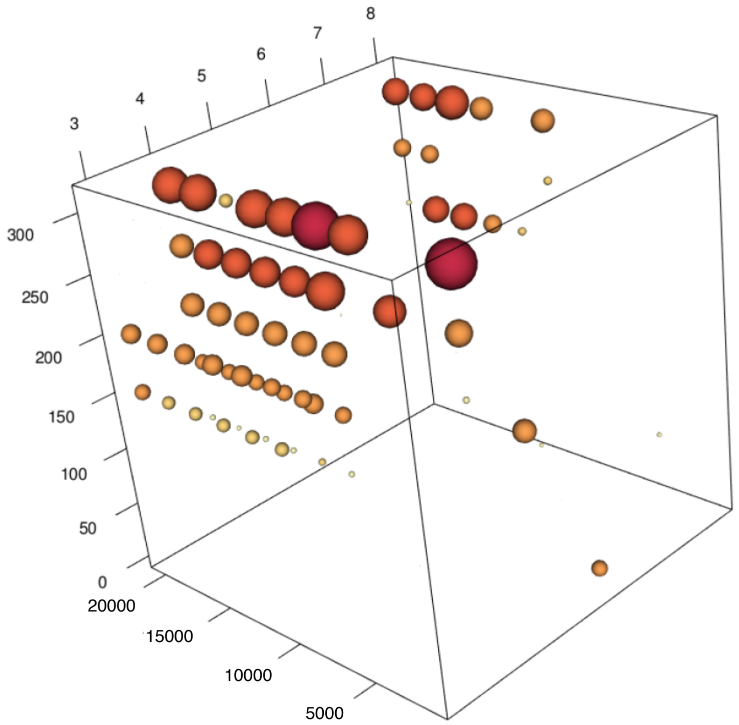

b.

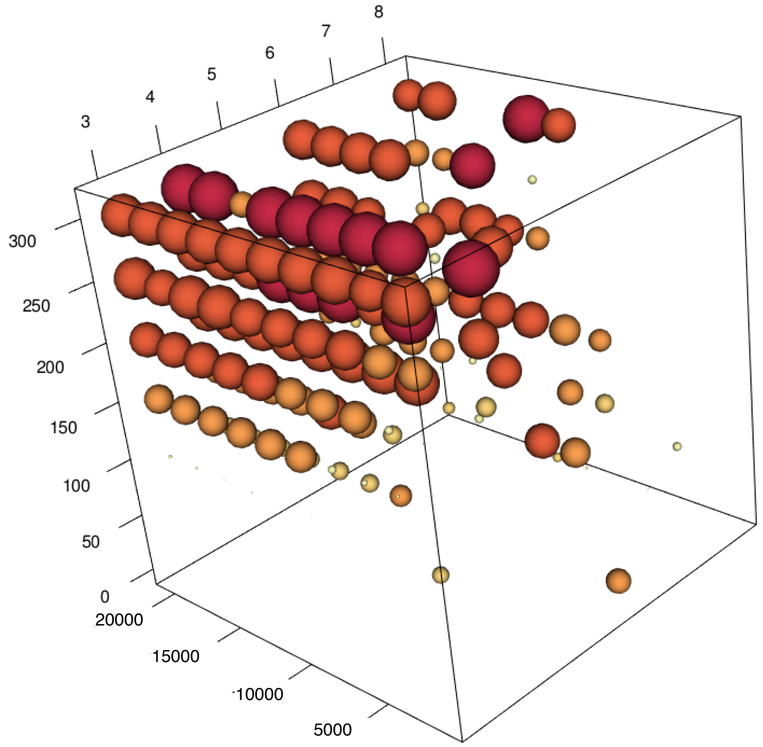

Supplementary Figure 6: Allelic spectra for non-monomorphic sites in the Platinum Genomes samples. Orange bars are the high-confidence calls from high-depth WGS, green bars are calls from downsampled BAMs at 1x. Differences in the shape of the distribution are likely due to the scarcity of information provided by the 1x data, which does not allow to recapitulate the family structure in the CEPH/Utah pedigree 1463, which composes the Platinum Genomes.

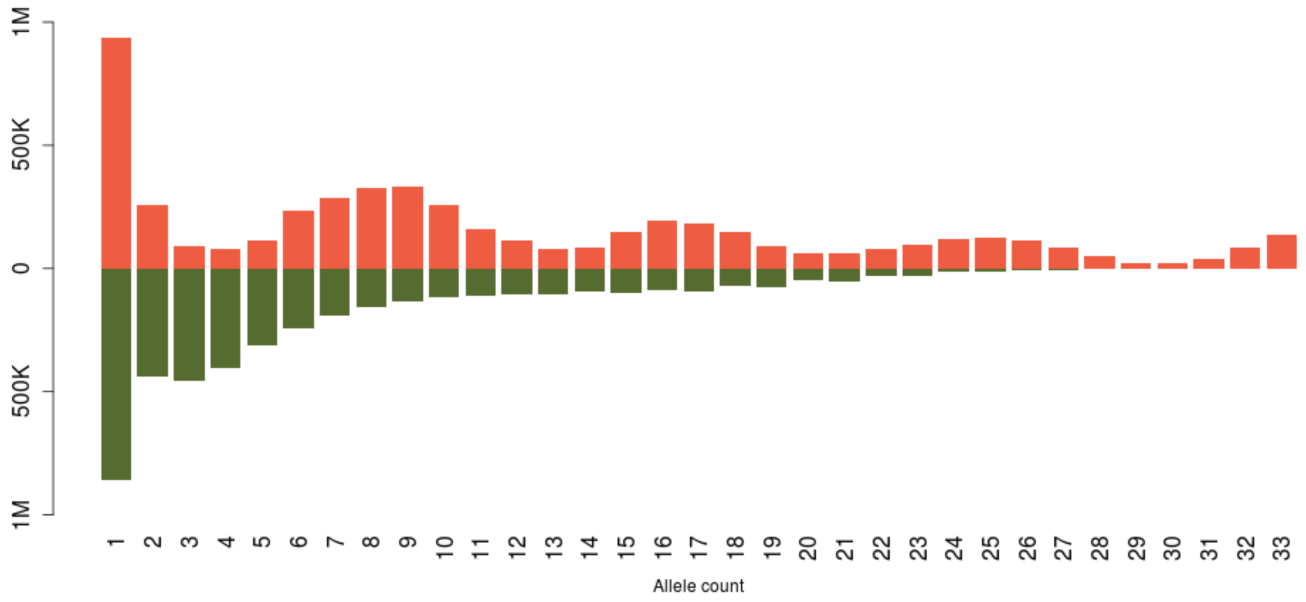

Supplementary Figure 7: Effect of decreasing chunk size and adding imputed variants on minor allele concordance and variant overlap. Concordance and overlap are evaluated against GWAS data. Purple bars: variant overlap in the refined-only dataset. Green bars: Imputed variants. Blue curve: Minor allele concordance, refined variants only using the 3-way reference panel, large chunk size (3,000 variants, 1000 flanking variants). Red curve: Refined variants only using the 3-way reference panel, small chunk size (1,500 variants, 500 flanking variants). Green curve: Imputed variants added, small chunk size. Black curve: Beagle phasing run added (no missingness).

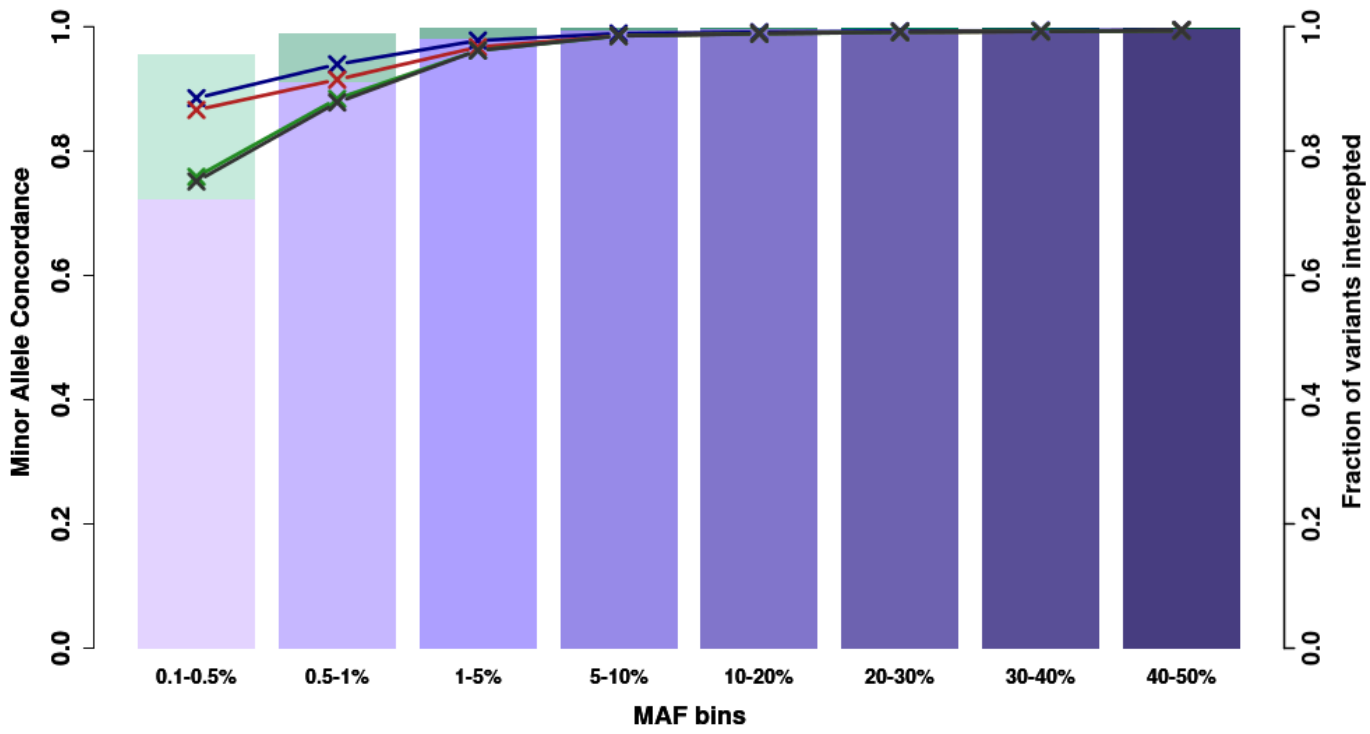

Supplementary Figure 8: Distributions and relationship between the two imputation accuracy measures provided by Beagle. Chromosome 11 data, imputed positions only. **a.** Correlation of Beagle imputation quality metrics. **b.** Distribution of Dosage R-squared. **c.** Allele frequencies of imputed variants. **d.** Dosage R-squared and allele frequency.

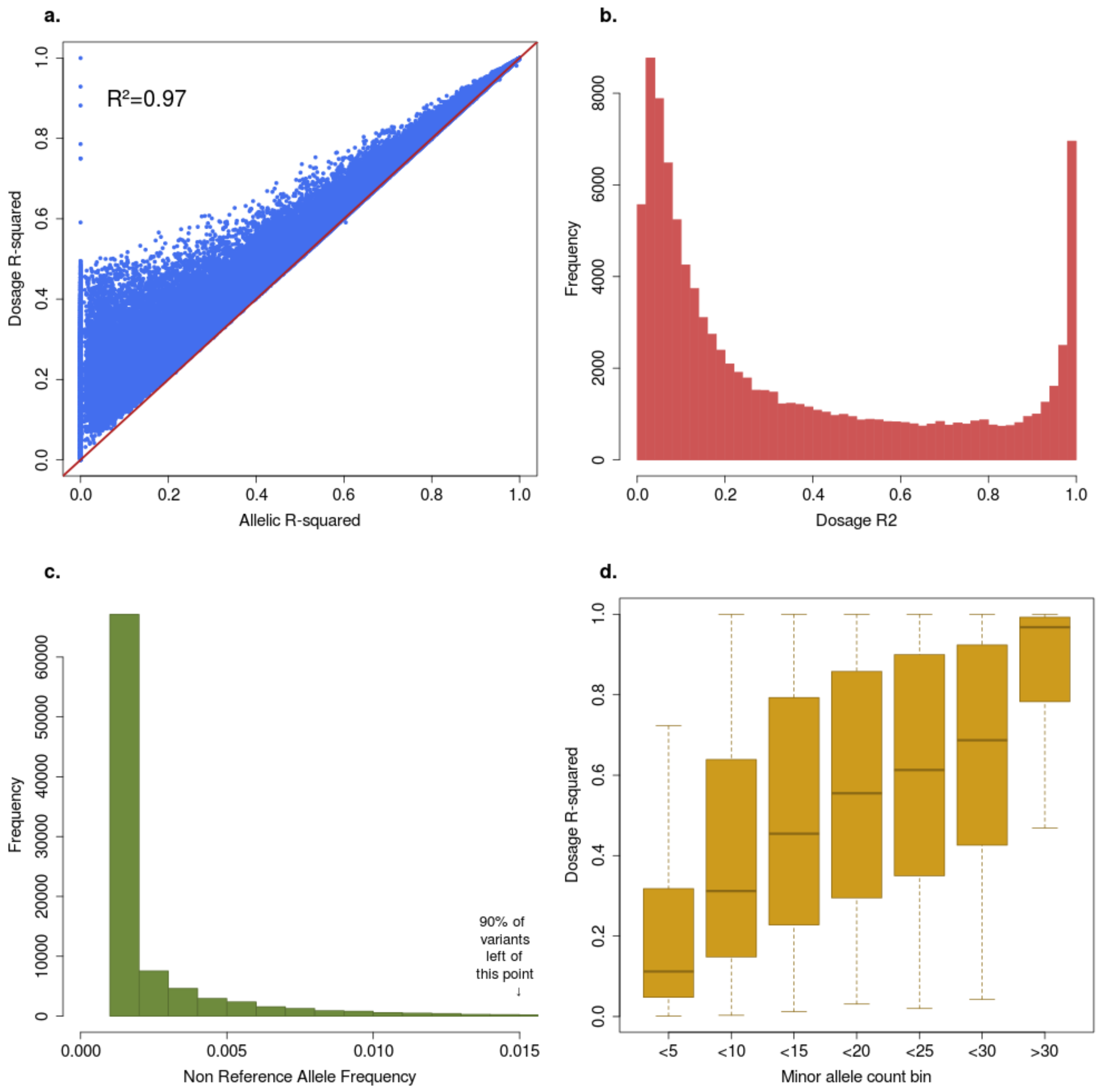

Supplementary Figure 9: Quality metrics for the 5 MANOLIS WES sequences. Metrics were extracted for chromosome 11. a. Variant quality score distribution. b. Call rate (AN) per quality score. c. Cumulative depth (DP) per quality score. d. Depth per sample. e. Quality per called alternative allele count (AC), on call-rate 100% SNVs only. Chosen thresholds are displayed in red where applicable.

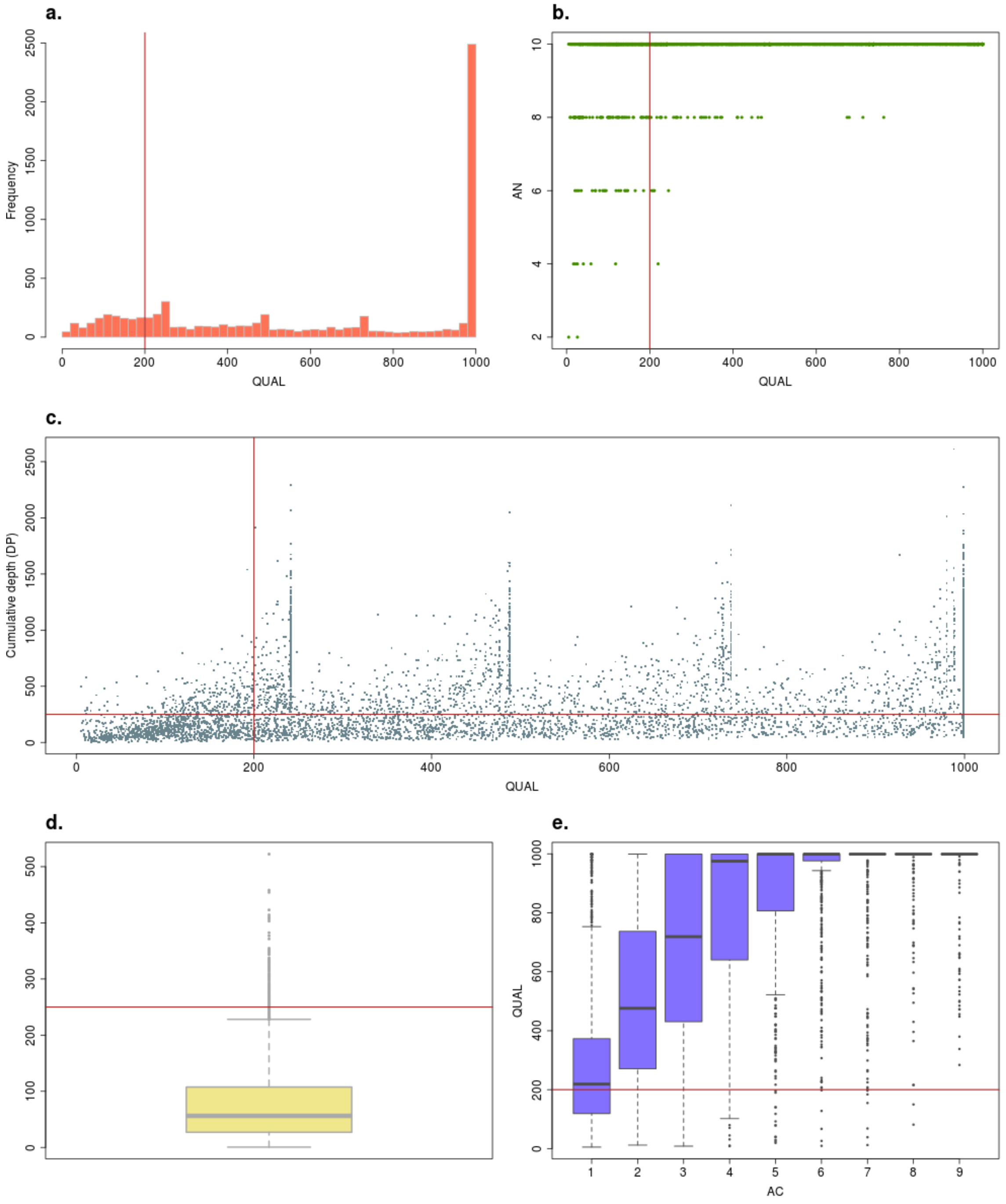

Supplementary Figure 10: Kinship coefficients have a limited effect on association statistics. **a.** Kinship coefficients in the genetic relatedness matrix are calculated using GEMMA using genome-wide data across 5 sets of sites, and are compared to the coefficients used in the analysis (red line, 20,667,092 sites, 1x WGS data, MAF>1%). All datasets are LD-pruned and filtered for Hardy-Weinberg equilibrium $p<1x10^{-5}$. **b.** QQ-plots of a triglycerides (TG) association using the highest (left) and lowest (right) kinship coefficients in **a**. The difference in lambda inflation factors is small (0.0172).

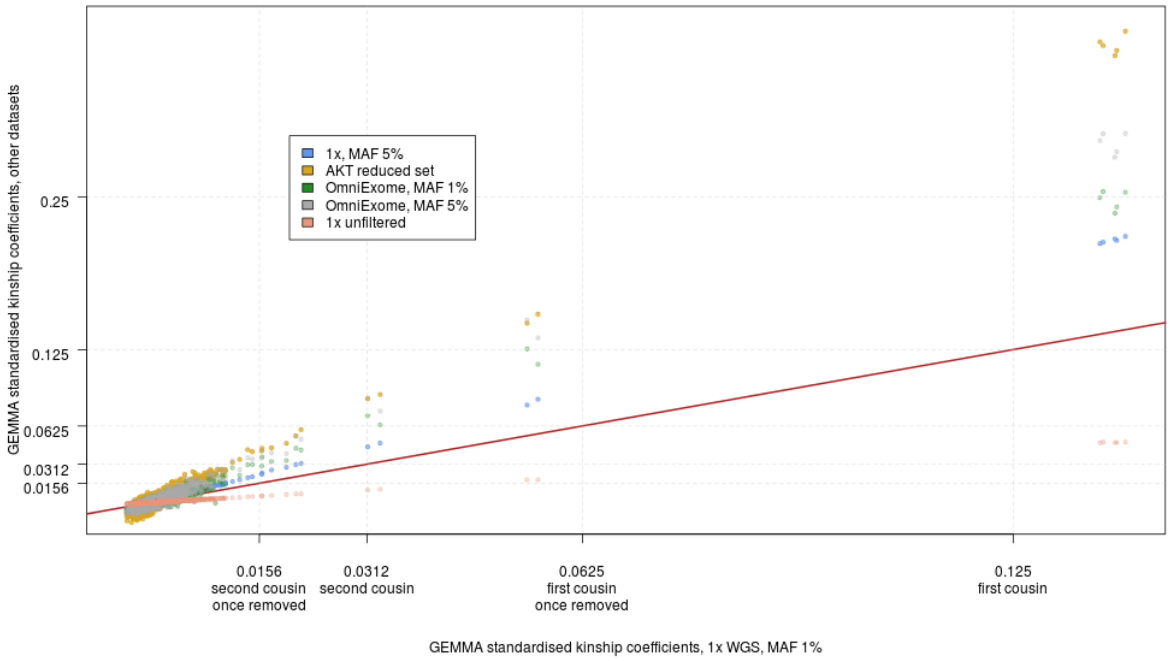

a.

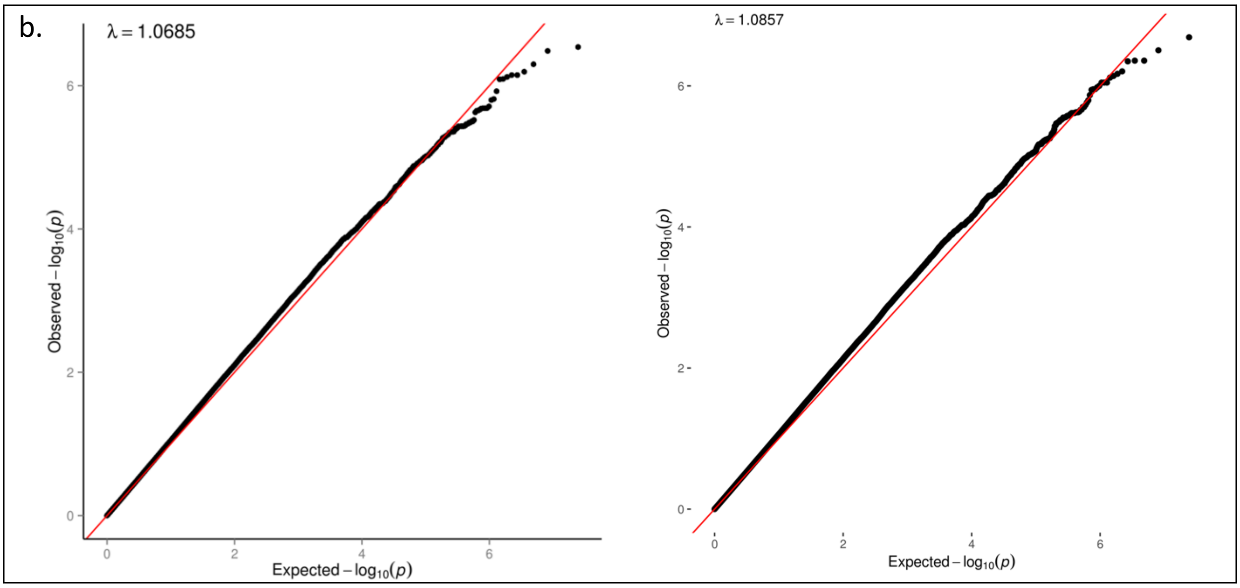

entary Tables

ntary Table 1: **Detail of the benchmarked genotype refinement and imputation pipelines.** Dashes indicate that no second
.

Name	First pass		Second pass	
	**Software**	**Reference Panel**	**Software**	**Reference Panel**
(1KG)	Beagle v.4	1000 Genomes Phase 1	-	-
(1KG) + Beagle 4	Beagle v.4	1000 Genomes Phase 1	Beagle v.4	None - phasing mode
(1KG) + IMPUTE2 (1KG)	Beagle v.4	1000 Genomes Phase 1	IMPUTE V2	1000 Genomes Phase 1
(1KG) + IMPUTE2	Beagle v.4	1000 Genomes Phase 1	IMPUTE V2	None – phasing mode
(1KG) + Beagle 4 (1KG)	IMPUTE V2	1000 Genomes Phase 1	Beagle v.4	1000 Genomes Phase 1
+ Beagle 4 (1KG)	IMPUTE V2	None - phasing mode	Beagle v.4	1000 Genomes Phase 1
(1KG)	IMPUTE V2	1000 Genomes Phase 1	-	-
(1KG) + Beagle 4	IMPUTE V2	1000 Genomes Phase 1	Beagle v.4	None – phasing mode
	Beagle v.4	None – phasing mode	-	-
+ IMPUTE2 (1KG)	Beagle v.4	None – phasing mode	IMPUTE V2	1000 Genomes Phase 1
	IMPUTE V2	None – phasing mode	-	-
+ Beagle 4	IMPUTE V2	None – phasing mode	Beagle v.4	None – phasing mode
+ IMPUTE2	Beagle v.4	None – phasing mode	IMPUTE V2	None – phasing mode

Supplementary Table 2: Lead variants in signals arising in the imputed GWAS (a) and the 1x WGS studies (b). Trait summaries and transformations are given in Supplementary Table 3. The effect size (beta) standard error (se), Wald, Likelihood Ratio and Score test values are from the GEMMA output. Variants are annotated using Ensembl via the REST API. Most severe consequence, rsID and mapped gene are obtained through Variant Effect Predictor. Previous associations are obtained through the Ensembl phenotype annotation overlap.

a. Imputed GWAS

trait	chr	ps	minor	major	MAF	beta	se	Wald test p-value	LR test p-value	score test p-value	previous associations	ensembl most severe consequence	rsID if applicable	mapped gene if applicable
LDL	2	22695702	A	G	0.008	1.21	0.24	4.90E-07	4.76E-07	6.35E-07	none	intron_variant	rs182210478	AC068490.2;AC096570.2
BGP	4	78130533	T	C	0.002	-2.58	0.51	4.05E-07	4.12E-07	5.92E-07	none	intron_variant	4:78130533	CCNG2
Fe_iron	2	182072184	C	CT	0.007	1.30	0.26	4.14E-07	4.04E-07	5.48E-07	none	intron_variant	chr2:182072184	AC104820.2
MCV	11	10720033	G	T	0.004	-1.95	0.38	3.11E-07	3.76E-07	7.36E-07	none	upstream_gene_variant	chr11:10720033	
RG	15	89987286	G	A	0.003	-2.16	0.408	1.07E-07	1.12E-07	1.69E-07	none	intergenic_variant	15:89987286	
RG	8	18632326	T	C	0.004	-1.97	0.36	5.80E-08	5.76E-08	8.73E-08	none	non_coding_transcript_exon_variant	rs76185967	PSD3;RPL35P6
RDW	11	10720033	G	T	0.006	-1.83	0.32	1.38E-08	1.35E-08	2.42E-08	none	upstream_gene_variant	chr11:10720033	
HDL	16	73298565	C	T	0.01	-1.37	0.22	4.91E-10	7.09E-10	1.83E-09	none	intergenic_variant	16:73298565	
BGP	16	62130142	C	A	0.015	0.86	0.17	4.17E-07	4.95E-07	7.82E-07	none	intergenic_variant	16:62130142	
LDL	14	72392076	A	C	0.016	-0.88	0.17	3.23E-07	3.60E-07	5.47E-07	none	intergenic_variant	14:72392076	
RG	11	62752440	A	G	0.014	-0.94	0.18	2.58E-07	2.59E-07	3.67E-07	none	5_prime_UTR_variant	11:62752440	SLC22A6
RG	17	10918336	A	C	0.027	0.71	0.13	8.25E-08	1.16E-07	2.27E-07	none	intergenic_variant	17:10918336	
HDL	3	4234628	A	C	0.023	0.79	0.14	2.44E-08	2.43E-08	3.79E-08	none	intron_variant	3:4234628	SUMF1
HGB	15	56731644	T	TA	0.043	0.67	0.12	1.41E-08	1.35E-08	2.41E-08	none	intron_variant	rs35812910	TEX9;MNS1
HCT	15	56731644	T	TA	0.043	0.69	0.12	6.24E-09	6.00E-09	1.14E-08	none	intron_variant	rs35812910	TEX9;MNS1
HDL	11	116701353	T	C	0.019	1.03	0.16	1.52E-10	1.65E-10	3.72E-10	HYPERALPHALIPOPROTEINEMIA 2	stop_gained;splice_region_variant	rs76353203	APOC3
HDL	16	66758967	T	C	0.018	-1.1	0.16	1.09E-11	1.41E-11	4.31E-11	none	intron_variant	chr16:66758967	DYNC1LI2;RP11-63M22.2
TG	11	116701353	T	C	0.019	-1.1	0.16	1.08E-11	1.16E-11	3.16E-11	HYPERALPHALIPOPROTEINEMIA 2	stop_gained;splice_region_variant	rs76353203	APOC3
TSH	2	228802502	G	A	0.336	-0.24	0.05	5.01E-07	4.91E-07	6.73E-07	none	intergenic_variant	2:228802502	
WHR	11	44108888	C	T	0.358	-0.24	0.05	4.17E-07	4.74E-07	7.62E-07	none	downstream_gene_variant	11:44108888	
WaistBMIadj	5	143052223	A	G	0.208	-0.29	0.06	3.40E-07	4.24E-07	7.39E-07	none	non_coding_transcript_exon_variant	5:143052223	CTB-57H20.1
FI	11	18054777	G	A	0.367	-0.28	0.05	3.66E-07	4.04E-07	7.11E-07	none	intron_variant	11:18054777	TPH1
adiponectinBMIadj	3	6390337	C	T	0.197	0.32	0.06	3.74E-07	3.70E-07	5.64E-07	none	intron_variant	rs1435723	AC026167.1
HGB	3	168885028	C	T	0.126	-0.37	0.07	3.25E-07	3.39E-07	5.27E-07	none	intron_variant	rs9809961	MECOM
LPCR	16	77938928	G	C	0.281	0.28	0.05	3.52E-07	3.38E-07	4.98E-07	none	intron_variant	16:77938928	VAT1L
WaistBMIadj	2	209253879	G	A	0.142	0.35	0.07	3.20E-07	3.09E-07	4.53E-07	none	intron_variant	2:209253879	PTH2R
MCHC	11	47377526	A	AC	0.314	-0.27	0.05	2.72E-07	2.62E-07	3.90E-07	none	double allele	rs142497874	SPI1
LDL	10	36066439	T	A	0.438	0.23	0.04	2.20E-07	2.50E-07	3.86E-07	none	intergenic_variant	rs10764055	
MID	16	81582440	C	G	0.22	-0.29	0.06	2.33E-07	2.24E-07	3.36E-07	none	intron_variant	16:81582440	CMIP
TC	20	16482169	T	C	0.118	0.34	0.06	1.77E-07	1.89E-07	2.84E-07	none	intron_variant	20:16482169	KIF16B
HipBMIadj	1	170198941	A	G	0.074	-0.48	0.09	1.68E-07	1.89E-07	3.22E-07	none	intron_variant	1:170198941	RP11-297H3.3
Height	8	116052374	G	C	0.096	0.42	0.08	1.48E-07	1.50E-07	2.34E-07	none	intergenic_variant	8:116052374	
LYM	17	77951023	T	C	0.257	0.29	0.06	1.41E-07	1.47E-07	2.37E-07	none	intron_variant	17:77951023	TBC1D16
TSH	10	91092558	A	T	0.239	0.27	0.05	1.46E-07	1.42E-07	2.02E-07	none	intron_variant	10:91092558	LIPA;IFIT3
TSH	3	193922574	T	G	0.146	-0.34	0.06	1.13E-07	1.20E-07	1.85E-07	none	intron_variant	3:193922574	RP11-513G11.4
LDL	19	45412079	T	C	0.079	-0.42	0.08	1.08E-07	1.07E-07	1.56E-07	Annotated by HGMD but no phenotype description is publicly available;Response to statins (LDL cholesterol change)	missense_variant;coding_sequence_variant	rs7412	APOE
RDW	12	127985024	C	A	0.051	-0.58	0.11	1.00E-07	1.03E-07	1.84E-07	none	intergenic_variant	12:127985024	
SGP	9	27869510	A	G	0.089	-0.4	0.07	8.65E-08	1.01E-07	1.68E-07	none	intergenic_variant	rs2383733	
adiponectin	4	73880050	C	G	0.202	-0.3	0.06	8.48E-08	8.93E-08	1.43E-07	none	intergenic_variant	rs62309628	
WBC	9	3624557	A	G	0.212	0.32	0.06	5.52E-08	8.04E-08	1.81E-07	none	intron_variant	9:3624557	RP11-509J21.1
SBPBMIadj	10	109676659	A	AG	0.486	-0.32	0.06	6.89E-08	6.75E-08	1.52E-07	none	intron_variant	rs112349650	RP11-215N21.1
TC	5	37915720	A	G	0.314	-0.25	0.05	6.87E-08	6.66E-08	9.72E-08	none	intron_variant	rs4574573	CTD-2194L12.2
HCT	3	168885028	C	T	0.126	-0.4	0.07	4.47E-08	4.75E-08	8.47E-08	none	intron_variant	rs9809961	MECOM
FI	4	72869003	C	A	0.339	-0.32	0.06	4.74E-08	4.52E-08	8.43E-08	none	intergenic_variant	4:72869003	
RDW	9	24017665	G	A	0.338	-0.29	0.05	4.38E-08	4.42E-08	7.45E-08	none	intergenic_variant	rs35456165	
RBC	10	131993	A	G	0.135	-0.39	0.07	3.30E-08	3.73E-08	6.83E-08	none	intron_variant	10:131993	IL9RP2
SBP	10	109676659	A	AG	0.481	-0.33	0.06	2.30E-08	2.32E-08	5.76E-08	none	intron_variant	rs112349650	RP11-215N21.1
RDW	17	54621347	C	T	0.096	-0.46	0.08	1.59E-08	1.54E-08	2.90E-08	none	intergenic_variant	17:54621347	
LDL	20	16482169	T	C	0.118	0.38	0.06	3.05E-09	3.41E-09	6.60E-09	none	intron_variant	20:16482169	KIF16B
TG	1	237565708	A	AT	0.179	0.33	0.06	3.05E-09	2.93E-09	5.05E-09	none	intron_variant	rs200918659	RYR2
HDL	16	57004889	A	G	0.417	0.33	0.04	8.72E-15	9.40E-15	4.74E-14	Triglycerides	intron_variant	rs7205804	CETP
Bilirubin	2	234664586	A	ATC	0.287	0.44	0.05	3.84E-20	5.21E-20	1.41E-18	none	non coding transcript exon variant	rs35754645	UGT1A8;UGT1A10;UGT1A9;UGT1A7;UGT1A6;UGT1A5;UGT1A4;UGT1A3

b. 1x WGS

trait	chr	ps	minor	major	MAF	beta	se	Wald test p-value	LR p-value	score test p-value	previous associations	ensembl most severe consequence	rsID if applicable	mapped gene if applicable
WHR	22	20747018	T	C	0.003	2.09	0.41	3.15E-07	3.24E-07	4.90E-07	none	upstream_gene_variant	rs1025878347	
HGB	11	8010293	G	A	0.007	-1.49	0.29	2.45E-07	2.44E-07	3.68E-07	none	intron_variant	rs11041700	EIF3F
adiponectin	18	55257281	T	C	0.006	-1.41	0.27	2.27E-07	2.27E-07	3.42E-07	none	upstream_gene_variant	rs75861770	
MCHC	4	161522850	T	C	0.006	-1.54	0.29	2.12E-07	2.04E-07	3.11E-07	none	intergenic_variant	rs75097797	
leptin	3	196163276	C	A	0.007	1.47	0.28	1.91E-07	1.84E-07	2.80E-07	none	upstream_gene_variant	novel	
Height	6	43216952	G	A	0.006	1.48	0.28	1.45E-07	1.41E-07	2.18E-07	none	intron_variant	novel	TTBK1
RG	8	18633903	G	T	0.003	-2.15	0.4	1.22E-07	1.24E-07	1.86E-07	none	intron_variant	rs978165175;rs142207507	PSD3
HDL	2	55373609	A	C	0.006	-1.42	0.27	1.22E-07	1.20E-07	1.73E-07	none	intergenic_variant	rs540995230	
Ferritin	6	71377781	A	C	0.006	-1.47	0.28	1.02E-07	1.02E-07	1.54E-07	none	missense_variant	rs112439957	SMAP1
RG	19	57109371	T	C	0.002	-2.41	0.44	6.15E-08	6.39E-08	1.00E-07	none	intron_variant	rs535491193	ZNF71
Height	1	95192620	A	G	0.009	-1.27	0.23	4.87E-08	4.86E-08	7.94E-08	none	intron_variant	rs145267153	LINC01057
gammaGT	4	3875226	A	G	0.009	1.23	0.22	4.40E-08	4.25E-08	6.42E-08	none	intergenic_variant	rs74608440	
adiponectinBMIadj	18	55257281	T	C	0.007	-1.63	0.28	1.03E-08	1.12E-08	2.50E-08	none	upstream_gene_variant	rs75861770	
HDL	16	64898622	G	T	0.004	-1.9	0.31	1.74E-09	1.67E-09	2.97E-09	none	intergenic_variant	novel	
TG	11	116701354	A	G	0.01	-1.33	0.2	7.43E-11	7.88E-11	1.74E-10	HYPERALPHALIPOPROTEINEMIA 2	splice_donor_variant	rs138326449	APOC3
HDL	16	69035884	C	A	0.008	-1.74	0.23	1.56E-13	1.48E-13	5.26E-13	none	intron_variant	rs370683912	TANGO6
HDL	16	70756259	T	C	0.007	-1.81	0.24	6.32E-14	5.99E-14	2.32E-13	none	intron_variant	rs188252530	VAC14
HDL	16	73298565	C	T	0.01	-1.6	0.21	1.46E-14	1.37E-14	6.06E-14	none	intergenic_variant	novel	
HDL	16	66378081	T	C	0.009	-1.79	0.22	1.10E-15	1.03E-15	5.86E-15	none	regulatory_region_variant	rs151205117	
Bilirubin	11	4437986	A	G	0.014	0.92	0.18	3.23E-07	3.16E-07	4.38E-07	none	upstream_gene_variant	rs148016671	
adiponectin	4	29843613	C	T	0.038	-0.57	0.11	3.13E-07	3.05E-07	4.32E-07	none	intergenic_variant	rs34891642	
TC	19	11388909	T	G	0.028	-0.63	0.12	2.79E-07	2.78E-07	3.92E-07	none	intergenic_variant	rs151018814	
PDW	2	197713430	C	T	0.037	0.61	0.12	2.89E-07	2.78E-07	4.11E-07	none	intron_variant	rs79078472	PGAP1
HDL	3	4234628	A	C	0.023	0.71	0.14	2.69E-07	2.71E-07	3.81E-07	none	intron_variant	rs138829006	SUMF1
LPCR	2	197713430	C	T	0.037	0.61	0.12	2.50E-07	2.41E-07	3.61E-07	none	intron_variant	rs79078472	PGAP1
leptin	19	32030022	A	T	0.015	-0.99	0.19	2.44E-07	2.39E-07	3.65E-07	none	upstream_gene_variant	rs142827020	
RDW	16	571731	A	G	0.011	-1.13	0.22	2.43E-07	2.36E-07	3.55E-07	none	3_prime_UTR_variant	rs183291067	RAB11FIP3
MID	3	108401373	G	A	0.05	-0.55	0.11	2.16E-07	2.08E-07	3.17E-07	none	intron_variant	rs116392621	DZIP3
BGP	18	26248392	T	A	0.046	-0.51	0.1	2.01E-07	1.95E-07	2.73E-07	none	intergenic_variant	rs112013002;rs1628183;rs771139413	
TC	14	72392076	A	C	0.016	-0.88	0.17	1.89E-07	1.84E-07	2.56E-07	none	intergenic_variant	novel	
Fe_iron	13	53763262	A	G	0.015	0.91	0.17	1.52E-07	1.48E-07	2.12E-07	none	intergenic_variant	rs116253332	
LYM	11	8059760	A	G	0.025	-0.79	0.15	1.14E-07	1.17E-07	1.91E-07	none	intron_variant	rs36206809	TUB;RP11-236J17.6
BGP	12	69338638	C	G	0.05	0.52	0.1	8.18E-08	8.59E-08	1.38E-07	none	intron_variant	rs117743406	CPM
TSH	19	22796761	T	C	0.035	-0.59	0.11	8.84E-08	8.56E-08	1.25E-07	none	downstream_gene_variant	rs35954710	
LDL	14	72392076	A	C	0.016	-0.9	0.17	7.84E-08	7.58E-08	1.10E-07	none	intergenic_variant	novel	
adiponectin	1	235746578	A	G	0.036	-0.6	0.11	7.33E-08	7.09E-08	1.09E-07	none	intron_variant	rs115072523	GNG4
RG	2	85968805	A	C	0.044	-0.55	0.1	6.94E-08	6.73E-08	9.93E-08	none	regulatory_region_variant	rs116010068	
TG	17	55839278	T	C	0.031	0.64	0.12	6.93E-08	6.71E-08	9.79E-08	none	regulatory_region_variant	rs9912215	
BGP	16	62130142	C	A	0.016	0.85	0.16	6.91E-08	6.68E-08	9.84E-08	none	intergenic_variant	rs28408198	
Height	15	26279796	A	G	0.014	-1.05	0.19	3.74E-08	3.71E-08	6.14E-08	none	intron_variant	rs189010705	RP11-1084I9.1
HGB	15	56719785	C	T	0.043	0.62	0.11	2.86E-08	2.73E-08	4.65E-08	none	intron_variant	rs553450981;rs795784;rs774475285	TEX9;MNS1
BGP	7	135326332	C	T	0.041	-0.59	0.1	2.75E-08	2.69E-08	4.18E-08	none	intron_variant	rs117784919	NUP205
MCV	16	571731	A	G	0.011	-1.28	0.23	2.37E-08	2.29E-08	4.01E-08	none	3_prime_UTR_variant	rs183291067	RAB11FIP3
FT4	5	19313119	C	A	0.021	0.78	0.14	1.75E-08	1.72E-08	2.77E-08	none	intergenic_variant	novel	
HCT	15	56719785	C	T	0.044	0.63	0.11	1.25E-08	1.19E-08	2.15E-08	none	intron_variant	rs553450981;rs795784;rs774475285	TEX9;MNS1
MCH	16	571731	A	G	0.011	-1.37	0.23	2.73E-09	2.60E-09	5.26E-09	none	3_prime_UTR_variant	rs183291067	RAB11FIP3
RG	11	62752440	A	G	0.013	-1.15	0.18	6.96E-10	6.73E-10	1.30E-09	none	5_prime_UTR_variant	rs57677322	SLC22A6
RG	17	10902383	G	C	0.027	0.81	0.13	1.97E-10	1.94E-10	4.15E-10	none	intergenic_variant	rs76050199	
HDL	11	116701353	T	C	0.014	1.24	0.17	3.30E-13	3.21E-13	1.10E-12	HYPERALPHALIPOPROTEINEMIA 2	stop_gained;splice_region_variant	rs76353203;CM086325	APOC3
TG	1	237565709	A	T	0.205	0.26	0.05	3.44E-07	3.34E-07	4.53E-07	none	intron_variant	rs753027291;rs12041884;rs200918659	RYR2
TG	2	134346210	C	T	0.289	-0.23	0.04	3.37E-07	3.27E-07	4.44E-07	none	intergenic_variant	rs6430433	
BMI	20	10411454	A	T	0.412	0.24	0.05	2.97E-07	3.00E-07	4.70E-07	none	intron_variant	rs6077785	MKKS
SGP	1	18197967	C	G	0.472	0.23	0.04	2.80E-07	2.72E-07	3.78E-07	none	intergenic_variant	rs618124	
WaistBMIadj	15	66483776	C	T	0.085	0.43	0.08	2.73E-07	2.71E-07	4.07E-07	none	intron_variant	rs28702433	MEGF11
HGB	5	34347349	G	A	0.376	0.24	0.05	2.81E-07	2.70E-07	4.01E-07	none	intergenic_variant	rs7726646	
BMI	14	81134882	T	C	0.099	-0.4	0.08	2.28E-07	2.69E-07	4.92E-07	none	intron_variant	rs114068807	CEP128
gammaGT	10	55148854	G	A	0.43	-0.22	0.04	2.44E-07	2.37E-07	3.29E-07	none	intergenic_variant	rs7082340	
FT4	13	103953533	A	G	0.059	0.47	0.09	2.43E-07	2.37E-07	3.30E-07	none	intergenic_variant	rs146017506	
TSH	3	193916181	A	G	0.205	-0.27	0.05	2.28E-07	2.21E-07	3.08E-07	none	downstream_gene_variant	rs59381142	
WaistBMIadj	11	44108888	C	T	0.343	-0.25	0.05	2.14E-07	2.09E-07	3.15E-07	none	downstream_gene_variant	rs7115682	
MPV	12	125082675	A	C	0.052	0.56	0.11	1.81E-07	1.81E-07	2.81E-07	none	regulatory_region_variant	rs12423958	
TC	2	101380915	G	A	0.285	-0.24	0.05	1.80E-07	1.81E-07	2.58E-07	none	intergenic_variant	rs6746018	
FI	11	18044648	G	A	0.356	-0.28	0.05	1.72E-07	1.64E-07	2.77E-07	none	non_coding_transcript_exon_variant	rs10741734	TPH1
PDW	12	125082675	A	C	0.052	0.56	0.11	1.60E-07	1.59E-07	2.47E-07	none	regulatory_region_variant	rs12423958	

MPV	19	8970903	T	C	0.101	-0.4	0.08	1.42E-07	1.59E-07	2.73E-07	none	intron_variant	rs4804087	MUC16
MPV	2	228619651	T	C	0.059	0.52	0.1	1.52E-07	1.52E-07	2.40E-07	none	intergenic_variant	rs62189960	
adiponectinBMIadj	7	25451594	A	T	0.393	-0.26	0.05	1.53E-07	1.52E-07	2.54E-07	none	intergenic_variant	rs6953343	
LPCR	2	228619651	T	C	0.059	0.52	0.1	1.47E-07	1.48E-07	2.34E-07	none	intergenic_variant	rs62189960	
Ferritin	15	33668251	G	T	0.067	0.43	0.08	1.51E-07	1.46E-07	2.08E-07	none	intron_variant	rs568235767	RYR3
TC	1	223480141	C	G	0.171	0.28	0.05	1.43E-07	1.40E-07	1.98E-07	none	intron_variant	rs1772271	SUSD4
HGB	3	168948198	C	T	0.11	-0.4	0.07	1.42E-07	1.37E-07	2.11E-07	none	intron_variant	rs75771628	MECOM
Fe_iron	7	105676297	G	A	0.073	-0.43	0.08	1.39E-07	1.34E-07	1.92E-07	none	3_prime_UTR_variant	rs144514220	CDHR3
PCT	20	840950	G	C	0.067	-0.48	0.09	1.25E-07	1.26E-07	2.00E-07	none	downstream_gene_variant	rs74531087	
PDW	3	86947782	A	T	0.299	-0.27	0.05	1.17E-07	1.14E-07	1.81E-07	none	intergenic_variant	rs9880801	
RDW	17	54621347	C	T	0.093	-0.42	0.08	1.03E-07	1.01E-07	1.59E-07	none	intergenic_variant	rs72835517	
HipBMIadj	1	170198941	A	G	0.072	-0.48	0.09	8.91E-08	9.63E-08	1.64E-07	none	intron_variant	rs7521954	RP11-297H3.3
TSH	10	91092706	C	T	0.242	0.26	0.05	9.91E-08	9.59E-08	1.39E-07	none	intron_variant	rs4630205	LIPA;IFIT3
MCV	10	36305596	C	T	0.141	-0.35	0.07	9.04E-08	8.69E-08	1.40E-07	none	intergenic_variant	rs3851069	
adiponectin	12	45861909	C	G	0.495	0.23	0.04	8.79E-08	8.46E-08	1.28E-07	none	intergenic_variant	rs4603367	
leptin	8	68464358	C	G	0.285	0.27	0.05	8.66E-08	8.33E-08	1.32E-07	none	intron_variant	rs924741	CPA6
FT4	7	152700215	G	A	0.187	0.29	0.05	8.11E-08	7.86E-08	1.15E-07	none	intergenic_variant	rs11486922	
LPCR	12	125082675	A	C	0.052	0.57	0.11	7.84E-08	7.72E-08	1.25E-07	none	regulatory_region_variant	rs12423958	
leptin	14	58047905	T	C	0.062	0.5	0.09	7.32E-08	7.31E-08	1.19E-07	none	non_coding_transcript_exon_variant	rs117786916	SLC35F4;RP11-409I10.2
MCHC	11	47393638	T	C	0.311	-0.27	0.05	7.57E-08	7.25E-08	1.17E-07	Fasting proinsulin	intron_variant	rs4992357	SPI1
FI	4	72869003	C	A	0.337	-0.31	0.06	7.33E-08	6.97E-08	1.25E-07	none	intergenic_variant	rs4263362	
TC	5	37915720	A	G	0.313	-0.24	0.04	6.56E-08	6.53E-08	9.70E-08	none	intron_variant	rs4574573	CTD-2194L12.2
Weight	3	193909907	T	C	0.24	0.29	0.05	5.34E-08	5.12E-08	8.15E-08	none	intergenic_variant	rs143707274	
LDL	19	45412079	T	C	0.086	-0.4	0.07	5.14E-08	4.98E-08	7.41E-08	Response to statins (LDL cholesterol change)	missense_variant;coding_sequence_variant	rs7412;CM860003	APOE
PLT	13	65366000	T	G	0.395	0.26	0.05	4.57E-08	4.49E-08	7.53E-08	Coronary Artery Disease	intergenic_variant	rs9540221	
RBC	16	434104	T	G	0.065	0.51	0.09	3.66E-08	4.23E-08	7.90E-08	none	intron_variant	rs72765895	TMEM8A;Z97634.3
HCT	3	168885028	C	T	0.127	-0.39	0.07	3.35E-08	3.20E-08	5.41E-08	none	intron_variant	rs9809961	MECOM
adiponectinBMIadj	3	6390337	C	T	0.195	0.34	0.06	2.79E-08	2.68E-08	4.78E-08	none	intron_variant	rs1435723	AC026167.1
TC	20	16492242	T	C	0.118	0.36	0.06	2.22E-08	2.15E-08	3.34E-08	none	intron_variant	rs6135772	KIF16B
HipBMIadj	6	33791230	T	C	0.346	0.27	0.05	1.87E-08	1.88E-08	3.54E-08	none	intergenic_variant	rs10947443	
Height	8	116052374	G	C	0.092	0.43	0.07	1.38E-08	1.40E-08	2.52E-08	none	intergenic_variant	rs71528491	
leptin	18	3312574	T	G	0.093	0.44	0.08	9.53E-09	9.12E-09	1.67E-08	none	intron_variant	rs7238627	RP11-838N2.5
SGP	9	27869510	A	G	0.085	-0.42	0.07	6.73E-09	6.80E-09	1.17E-08	none	intergenic_variant	rs2383733	
MPV	3	86947782	A	T	0.299	-0.29	0.05	6.09E-09	5.81E-09	1.10E-08	none	intergenic_variant	rs9880801	
MIDPC	14	103817631	C	T	0.34	0.29	0.05	4.80E-09	4.61E-09	8.88E-09	none	intergenic_variant	rs2403168	
SBPBMIadj	10	109678540	A	G	0.506	0.34	0.06	4.72E-09	4.50E-09	1.35E-08	none	intron_variant	rs982601001;rs10884572	RP11-215N21.1
LDL	10	36066439	T	A	0.441	0.25	0.04	2.99E-09	2.91E-09	5.12E-09	none	intergenic_variant	rs10764055	
WBC	9	3624557	A	G	0.212	0.34	0.06	1.35E-09	1.28E-09	2.77E-09	none	intron_variant	rs58276821	RP11-509J21.1
SBP	10	109678540	A	G	0.51	0.35	0.06	1.18E-09	1.13E-09	3.85E-09	none	intron_variant	rs982601001;rs10884572	RP11-215N21.1
LDL	20	16482169	T	C	0.119	0.39	0.06	7.80E-10	7.51E-10	1.41E-09	none	intron_variant	rs76841054	KIF16B
WaistBMIadj	5	143075283	G	A	0.208	-0.34	0.05	4.87E-10	4.63E-10	1.04E-09	none	intron_variant	rs11749182	CTB-57H20.1
RBC	10	124767	T	C	0.138	-0.42	0.06	9.16E-11	9.20E-11	2.47E-10	none	upstream_gene_variant	rs9419461	
HDL	16	57005479	A	C	0.417	0.35	0.04	2.66E-17	2.51E-17	2.15E-16	Triglycerides	intron_variant	rs1532624	CETP
Bilirubin	2	234673309	T	C	0.287	0.45	0.04	1.53E-22	1.62E-22	8.45E-21	Total bilirubin levels in HIV-1 infection	intron_variant	rs4148325	UGT1A8;UGT1A10;UGT1A9;UGT1A7;UGT1A6;UGT1A5;UGT1A4;UGT1A3;UGT1A1

Supplementary Table 3: Independent signals across MAF categories in the 1x and imputed GWAS, before and after filtering for associations significant at 5x10-5 in the 22x WGS dataset.

		rare	low-frequency	common
**post-filtering**	**1x**	19	30	59
	**imputed GWAS**	8	10	14
	**ratio**	**2.3**	**3**	**4.2**
**pre-filtering**	**1x**	56	35	91
	**imputed GWAS**	130	118	214
	**ratio**	**2.3**	**3.4**	**2.3**

Supplementary Table 4: Phenotypes used in the association analysis.

Trait name	Description	Unit	Transformation	covariates	exclusion
adiponectinBMIadj	adiponectin adjusted for BMI	ug/mL	inverse normal	age, age^2, BMI	+/- 5 S.D
adiponectin	adiponectin	ug/mL	inverse normal	age, age^2	+/- 5 S.D
BGP	osteocalcin	mg/ml	inverse normal	age, age^2	+/- 5 S.D
Bilirubin	bilirubin	mg/dl	inverse normal	age, age^2	+/- 5 S.D
BMI	Body Mass Index	kg/m^2	inverse normal	age, age^2	>4 S.D
CRP	C-reactive protein	mg/L	inverse normal	age, age^2	+/- 3 S.D and <0.1, >10
DBPBMIadj	diastolic blood pressure adjusted for BMI	mmHg	inverse normal	age, age^2, BMI	+/- 5 S.D
Fe_iron	Iron	mmol/L	inverse normal	age, age^2	+/- 5 S.D
Ferritin	Ferritin	pmol/L	inverse normal	age, age^2	+/- 5 S.D
FG	Fasting glucose	mmol/L	inverse normal	age, age^2	>7
FIBMIadj	Fasting insulin adjusted for BMI	uIU/ml	inverse normal	age, age^2, BMI	+/- 5 S.D
FI	Fasting insulin	uIU/ml	inverse normal	age, age^2	+/- 5 S.D
FT4	Free Thyroxine	ng/dl	inverse normal	age, age^2	+/- 5 S.D
gammaGT	gamma-glutamyltransferase	IU/L	inverse normal	age, age^2	+/- 5 S.D
GRAN	granulocytes	NA	inverse normal	age, age^2	none
GRANPC	granulocytes, percent	NA	inverse normal	age, age^2	none
HCT	haematocrit	%	inverse normal	age, age^2	+/- 3 S.D
HDL	high-density lipoprotein	mmol/L	inverse normal	none	+/- 5 S.D
Height	height	cm	inverse normal	age, age^2	+/- 5 S.D
HGB	haemoglobin	g/dl	inverse normal	age, age^2	+/- 5 S.D
HipBMIadj	hip circumference adjusted for BMI	cm	inverse normal	age, age^2, BMI	>4 S.D
Hip	hip circumference	cm	inverse normal	age, age^2	>4 S.D
HOMA_bBMIadj	homeostatic model assessment, b-cell function, adjusted for BMI	-	inverse normal	age, age^2, BMI	none
HOMA_b	homeostatic model assessment, b-cell function	-	inverse normal	age, age^2	none
HOMA_irBMIadj	homeostatic model assessment, insulin resistance, adjusted for BMI	-	inverse normal	age, age^2, BMI	none
HOMA_ir	homeostatic model assessment, insulin resistance	-	inverse normal	age, age^2	none
LDL	low-density lipoprotein	mmol/L	inverse normal	none	+/- 5 S.D
leptin	leptin	ng/mL	inverse normal	age, age^2	+/- 5 S.D
LPCR	large platelet concentration ratio	-	inverse normal	age, age^2	none
LYM	lymphocytes	-	inverse normal	age, age^2	none
LYMPC	lymphocytes, percent	%	inverse normal	age, age^2	none
MCHC	mean corpuscular haemoglobin concentration	g/dL	inverse normal	age, age^2	+/- 3 S.D
MCH	mean content of haemoglobin	pg	inverse normal	age, age^2	+/- 3 S.D
MCV	mean corpuscular volume	fl	inverse normal	age, age^2	+/- 3 S.D
MID	monocytes, eosinophils, basophils	-	inverse normal	age, age^2	none
MIDPC	monocytes, eosinophils, basophils, percent	-	inverse normal	age, age^2	none
MPV	mean platelet volume	-	inverse normal	age, age^2	none
PCT	plateletcrit	-	inverse normal	age, age^2	none
PDW	platelet distribution width	-	inverse normal	age, age^2	none
PLT	platelets	10^9/L	inverse normal	age, age^2	+/- 3 S.D
RBC	red blood cell count	10^12/L	inverse normal	none	+/- 3 S.D
RDW	red cell distribution width	-	inverse normal	age, age^2	none
RDWPC	red cell distribution width, percent	-	inverse normal	age, age^2	none
RG	random glucose	mmol/L	inverse normal	age, age^2	none
RI	random insulin	uIU/ml	inverse normal	age, age^2	none
SBPBMIadj	systolic blood pressure, adjusted for BMI	mmHg	inverse normal	age, age^2, BMI	+/- 5 S.D
SBP	systolic blood pressure	mmHg	inverse normal	age, age^2	+/- 5 S.D
SGP	alanine aminotransferase	IU/L	inverse normal	age, age^2	+/- 5 S.D
TC	total cholesterol	mmol/L	inverse normal	none	+/- 5 S.D
TG	triglycerides	mmol/L	log	none	+/- 5 S.D
TSH	thyroid stimulating hormone	uIU/ml	inverse normal	age, age^2	+/- 5 S.D
WaistBMIadj	waist circumference adjusted for BMI	cm	inverse normal	age, age^2, BMI	>4 S.D
Waist	waist circumference	cm	inverse normal	age, age^2	>4 S.D
WBC	white blood cells	10^9/L	log	age, age^2	+/- 3 S.D
Weight	weight	kg	inverse normal	age, age^2	>4 S.D
WHRBMIadj	waist-hip ratio adjusted for BMI	-	inverse normal	age, age^2, BMI	>4 S.D
WHR	waist-hip ratio	-	inverse normal	age, age^2	>4 S.D

Supplementary Text

Read alignment and calling

Following generation of raw reads on the Illumina HiSeq 2000 and HiSeq 2500 sequencing machines, reads were converted from BCL format to BAM format using the Illumina2BAM (https://github.com/wtsi-npg/illumina2bam) software. Illumina2BAM was again used to de-multiplex lanes that had been sequenced so that the tags were isolated from the body of the read, decoded, and could be used to separate out each lane into lanelets containing individual samples from the multiplex library and the PhiX control. The quality scores were then recalibrated using the purity recalibration algorithm (Abnizova, et al., 2010) using the PhiX data for reference. Read mapping was then carried out using the BWA backtrack algorithm version 0.5.10 using the GRCh37 1000 Genomes phase III reference (also known as hs37d5). PCR and optically duplicated reads were marked using Picard MarkDuplicates (http://broadinstitute.github.io/picard).

In order to ensure the quality of the large quantity of BAMs produced for the project, an automatic quality control system was used to reduce the number of data files that required manual intervention. This system was derived from the one originally designed for the UK10K project (http://www.uk10k.org) and used a series of empirically derived thresholds to assess summary metrics calculated from the input BAMs. These thresholds included: percentage of reads mapped; percentage of duplicate reads marked; various statistics measuring INDEL distribution against read cycle and an insert size overlap percentage. Any lane that fell below the “fail” threshold for any of the metrics were excluded; and any lane that did not fall below these thresholds for any of the metrics was given a status of “pass” and allowed to proceed into the later stages of the pipeline.

Passed lanelets were then merged into BAMs corresponding to the libraries for each sample and duplicates were marked again with Picard MarkDuplicates after which they were then merged into BAMs on a per sample basis. Finally sample level bam improvement was carried out using GATK 1.6(DePristo, et al., 2011; McKenna, et al., 2010) and samtools(Christopoulos, 1997) from git commit 72d6457f7f361c323f62bd2d3170980132ba2113. This consisted of re-alignment of reads around known and discovered INDELs followed by base quality score recalibration both using the GATK, lastly samtools calmd was applied and indexes were created. Known INDELs for realignment were taken from Mill Devine and 1000G Gold set and the 1000G phase low coverage set both part of the Broad’s GATK resource bundle version 2.2. Known variants for BQSR were taken from dbSNP 137 also part of the Broad’s resource bundle. The input BAM files are fed into samtools mpileup to create all-sites BCF files, which are piped into bcftools view to create variant-only VCF files containing genotype calls. We split the genome into chunks of 100,000 base pairs, and separate these chunks into SNV and INDEL files. We run GATK UnifiedGenotyper to calculate site-level annotations.

Variant Quality Score Recalibration

Variant quality score recalibration was performed using the QualByDepth, HaplotypeScore, MQRankSum, ReadPosRankSum, FS, InbreedingCoeff and DP annotations. Initial runs were performed on GATK v. 2.7.2, final runs were performed using GATK 3.1.1. With identical input, this change in VQSR version had large consequences in the recalibration output (Supplementary Figure 3). For completeness, we tested recalibration using GATK v.3.6 at the

time of publication, which uses a different set of recommended annotations (HaplotypeScore was removed from the model, and MQ was added). This yielded very poor performance for the previously optimal run, with an increasing Ti/Tv by tranche curve instead of a strictly decreasing one. We then restored the set of annotations to HaplotypeScore, BaseQRankSum, MQRankSum, ReadPosRankSum, FS and InbreedingCoeff and obtained a very good performance, with a 99.4% sensitivity at the 2.0 Ti/Tv cutoff point, while conserving the 10% type-I error rate. This is a much better sensitivity/specificity compromise than the cut-off chosen at the analysis stage of this work, and highlights the extreme variability of recalibration results for very-low-depth sequencing data.

Common-variant 1x signals not detected using GWAS and imputation
As shown in our comparison, 1x WGS detects at least twice, and up to four times as many true common variant signals than imputed GWAS (Supplementary Table 1). We do not expect this is due to higher genotype quality, as imputation is reliable for common variants. If this were true, high-depth WGS p-values would be close to 1x ones, however we observe instead an almost systematic overestimation of association significance. We posit this is due to lower genotype quality. Indeed, if due to a noisier input, genotypes are more inaccurate in the 1x imputation, we expect more genotyping error and hence more variability around the association p-value we would get with perfect genotypes. By selecting for low p-values in our comparison, we are biasing against variants for which small genotyping errors lead to an underestimation of the effect of genotype on phenotype. As shown in Figure , most of the signals confirmed in the 15x data do have a lower p-value in the 1x study than in both the 22x and imputed GWAS, confirming this hypothesis. This slightly decreased imputation accuracy also explains why we see more successfully detected common variant signals in the 1x and the 15x compared to the imputed GWAS. This does not put in question our findings regarding the power of 1x for detecting association, but it does indicate a slightly lower accuracy, which incidentally remains very high for common variants (Figure 2).

References

Abnizova, I.*, et al.* Statistical comparison of methods to estimate the error probability in short-read Illumina sequencing. *J Bioinform Comput Biol* 2010;8(3):579-591.

Christopoulos, K.T.D. Minorities in Greece. Kritiki; 1997.

DePristo, M.A.*, et al.* A framework for variation discovery and genotyping using next-generation DNA sequencing data. *Nat Genet* 2011;43(5):491-498.

McKenna, A.*, et al.* The Genome Analysis Toolkit: a MapReduce framework for analyzing next-generation DNA sequencing data. *Genome Res* 2010;20(9):1297-1303.
